# Urinary Biomonitoring and Risk Prioritization of Traditional and Emerging Neonicotinoids in School-Aged Children, South China

**DOI:** 10.3390/toxics14080735

**Published:** 2026-08-21

**Authors:** Pan Zhu, Ling-Chuan Guo, Xuan Liu, Junwei Yang, Guangning Su, Jing Deng, Xiuhua Zhong, Chaoyang Long, Jingguang Li, Shengbing Yu

**Affiliations:** 1 Guangdong Provincial Center for Disease Control and Prevention, Guangzhou 511430, China; zhupan903@126.com (P.Z.);; 2 State Key Laboratory of Environmental Criteria and Risk Assessment, State Environmental Protection Key Laboratory of Ecological Effect and Risk Assessment of Chemicals, Chinese Research Academy of Environmental Sciences, Beijing 100012, China; 3 Guangdong-Hong Kong-Macao Joint Laboratory for Contaminants Exposure and Health, Guangdong Key Laboratory of Environmental Catalysis and Health Risk Control, Institute of Environmental Health and Pollution Control, Guangdong University of Technology, Guangzhou 510006, China; 4 China National Center for Food Safety Risk Assessment, Beijing 100012, China

**Keywords:** neonicotinoids, metabolites, school-aged children, risk assessment, cumulative exposure, toxicological prioritization

## Abstract

The widespread use of neonicotinoids (NEOs) poses a significant risk to school-aged children. However, critical knowledge gaps remain regarding their comprehensive exposure profiles and associated health risks. This study assessed the co-exposure levels and non-carcinogenic risks of 16 NEOs (including both traditional and emerging compounds) in 184 school-aged children recruited from a typical rural and urban area of South China. The sum concentrations of 16 NEOs (∑_16_NEOs) ranged from 0.648 to 227 μg/L (median: 8.99 μg/L). The predominant compounds were clothianidin (CLO, 1.93 μg/L), *N*-desmethyl-acetamiprid (*N*-dm-ACE, 1.64 μg/L), thiamethoxam (THM, 0.792 μg/L), and dinotefuran (DIN, 0.222 μg/L). To our knowledge, this is the first report of emerging NEOs—paichongding (IPP), sulfoxaflor (SUL), and flonicamid (FLO)—in children urine, with detection frequencies of 11.4%, 68.2%, and 53.4%, respectively. Significant rural–urban disparities in urinary concentrations were observed. The metabolite-to-parent ratios (e.g., *N*-dm-ACE/ACE, 5-OH-IMI/IMI, and THCP-AM/THCP) may serve as a useful biomarker for assessing human exposure to NEOs, as it provides valuable insight into the metabolic fate of the target compounds and aids in distinguishing exposure pathways. The median estimated daily intake (EDI) values for CLO, *N*-dm-ACE, THM, and ∑_12_NEOs were 0.127, 0.117, 0.046, and 0.696 μg/kg-bw/day, respectively. Although the hazard quotient for individual compounds was below 1 for vast majority of school-aged children, cumulative exposure assessment indicated that 1.09% of participants had a hazard quotient exceeding 1 for both IMI_eq_ and ∑_12_NEOs, suggesting potential health concerns under co-exposure scenarios. Using a multi-criteria Toxicological Priority Index (ToxPi) model, CLO, THM, thiacloprid-amid (THCP-AM), and *N*-dm-ACE were identified as the high-priority compounds requiring regulatory attention in children’s environmental health. This study provides the first comprehensive biomonitoring and ToxPi-based prioritization of both traditional and emerging NEOs in school-aged children in South China. The findings highlight the urgent need for continued monitoring, refined mixture risk assessment, and regulatory consideration of emerging substitutes and their metabolites, which are not adequately covered by current safety guidelines.

## 1. Introduction

Neonicotinoids (NEOs), a new generation of systemic insecticides, remain the most extensively used class of insecticides globally, accounting for approximately 27% of the world market [1]. The cost-effectiveness, high insecticidal efficacy, and the relatively slow development of resistance in target pests [2] have led to their intensive application across agricultural, industrial, and residential environments. In particular, non-agricultural sources, including residential biocide application and veterinary medicine use, have been increasingly recognized as important contributors to human NEO exposure [3]. Acetamiprid (ACE) and imidacloprid (IMI) are insecticides of global importance and are often used as spray and watering agents for ornamental plants and veterinary medication to control biting and sucking insects or as topical medications on pets to remove and control fleas [3]. The intensive application of NEOs has led to widespread environmental contamination and mounting evidence of health risks, including reproductive toxicity, cardiotoxicity, genotoxicity, hepatotoxicity, neurotoxicity, and endocrine-disruption effects [4,5,6,7].

In response to the non-target adverse effects of NEOs, regulatory actions have been implemented worldwide, though the scope and stringency vary considerably. The European Union has adopted the most restrictive approach, banning the outdoor use of IMI, clothianidin (CLO), and thiamethoxam (THM), and has scheduled a ban on imports of products containing THM and CLO by 2026 [8]. In China, NEOs remain approved for both agricultural and certain non-agricultural uses, including residential insect control and veterinary ectoparasiticides, although the regulatory framework differs substantially from that in the EU and North America. In North America, regulatory measures are more fragmented. Since 1 January 2026, Washington State has prohibited the use of ACE, CLO, dinotefuran (DIN), IMI, nitenpyram (NIT), and THM, on non-productive outdoor ornamental plans, trees, and lawns [9]. Meanwhile, two emerging NEOs, sulfoxaflor (SUL) and flupyradifurone (FLU), are currently undergoing approval renewal reviews as of 2025, signaling a shifting regulatory landscape for next-generation compounds.

To date, regulatory actions have mainly targeted a limited set of traditional NEOs—DIN, ACE, IMI, thiacloprid (THCP), THM, and CLO. This regulatory pressure has gradually driven the adoption of emerging substitutes such as, SUL, FLU, cycloxaprid (CYC), imidaclothiz (IMIZ), paichongding (IPP) and flonicamid (FLO) [10]. These alternatives are now used extensively, for example, a 2019 survey in Guangdong Province, China, reported annual usage of 62.0 metric tons of NIT, 28.0 metric tons of IMIZ, and 14.0 metric tons of FLO, with agriculture accounting for 83.1% of total use. Consequently, these emerging compounds have been detected in various environmental matrices, including the Pearl River [11], wastewater treatment plants [12], powdered green tea, and plant-derived foodstuffs [13]. However, despite clear evidence of their environmental occurrence, data on human exposure to these emerging substitutes remain very limited. Therefore, it is urgent to investigate whether these emerging NEOs pose a greater threat to human health than the traditional NEOs.

The widespread occurrence of NEOs has been demonstrated by their ubiquitous detection in urine across diverse populations, including pregnant women, adults, children, and neonates [4,14,15,16]. Following ingestion, parent NEOs (p-NEOs) are subjected to cytochrome P450-mediated metabolism, yielding metabolites (m-NEOs) such as *N*-desmethyl-acetamiprid (*N*-dm-ACE), thiacloprid-amid (THCP-AM) and 5-hydroxy-imidacloprid (5-OH-IMI) [17]. Notably, these m-NEOs often exhibit greater persistence than their parent compounds, leading to higher detection frequencies and concentrations in urine biomonitoring studies [18,19]. Moreover, several m-NEOs have been shown to exhibit greater toxicity relative to p-NEOs [18]. For instance, cross-sectional studies have associated *N*-dm-ACE with impaired insulin-glucose homeostasis, and 5-OH-IMI has been linked to reduced serum testosterone levels and compromised semen quality [20]. Of note, *N*-dm-ACE has been reported as the predominant analyte in both maternal and cord serum [21]; its concentration appears to be inversely associated with the risk of general obesity [22].

Despite these findings, a critical methodological gap remains. To date, most biomonitoring studies have focused on either a limited set of traditional NEOs (DIN, ACE, IMI, THCP, THM, and CLO, NIT) or their metabolites in isolation, and few have simultaneously quantified the full spectrum of traditional compounds alongside emerging substitutes within the same population. Recent analytical advancements have demonstrated the feasibility of simultaneously detecting multiple NEOs and metabolites in human urine (e.g., 21 NEOs and metabolites in a single assay) [23], yet the simultaneous determination of both traditional and emerging NEOs in vulnerable populations such as school-aged children remains scarce. This lack of comprehensive coverage has hampered the ability to characterize the true exposure landscape and to compare the relative contributions of traditional and emerging compounds to total internal exposure.

Due to variations in living environments, dietary habits, and metabolic rates, children exhibit distinct exposure profiles compared with adults [24,25]. Children have higher estimated daily intake (EDI) values than adults; their exposure profile can be modulated by daily activity, dietary selection, and nearness to agricultural operations [4]. Despite these recognized determinants, the combined influence of dietary habits, residential environment, and demographic factors on the exposure profiles of both traditional and emerging NEOs in school-aged children has not been systematically characterized. Current knowledge on the NEO-related health burden in children remains remarkably limited both in terms of scope and the number of chemicals assessed [24,26]. Specifically, while traditional NEOs such as CLO, THM, DIN, ACE, THCP, and IMI have received some scientific attention, their metabolic derivatives (e.g., *N*-dm-ACE, THCP-AM, 5-OH-IMI) and emerging substitutes remain critically understudied. Furthermore, school-aged children are in a fragile window: they have insufficient detoxification capacity due to immature organ systems, receive higher exposure doses per unit body weight, exhibit distinct behavioral exposure patterns, and are more sensitive to irreversible damage sensitivity during critical neurodevelopmental windows than adults [27]. These characteristics may lead to significant health effects even under low-level, chronic exposures. For this reason, NEO exposure in children is of particular concern, and more stringent safety factors and exposure assumptions must be investigated to protect their health.

In addition to the gaps in exposure characterization and source identification, a further challenge lies in how to translate the accumulating exposure data into actionable chemical management strategies. Traditional risk assessment approaches often rely on a single metric such as hazard quotient or margin of exposure. This risk assessment method may not adequately capture the complexity of multi-chemical exposure scenarios. The Toxicological Priority Index (ToxPi) model, originally developed by [28], is a flexible, data-driven multi-criteria framework that enables the systematic integration of multiple data streams for chemical prioritization. As a data-driven, multi-criteria framework, the ToxPi model moves beyond concentration-based assessments by effectively aggregating and visualizing information from diverse toxicity endpoints, persistence, and exposure. In recent years, the ToxPi model has gained increasing application in environmental chemical prioritization. For instance, studies have employed ToxPi to screen 112 priority emerging contaminants [29] and rank neurotoxicity-informed assessment of psychoactive substances in the Yangtze River [30]. However, to the best of our knowledge, the ToxPi model has not yet been applied to prioritize NEOs in the context of human biomonitoring, particularly for the purpose of identifying which compounds—whether traditional or emerging, parent or metabolite—warrant prioritized control from a children’s health protection perspective. Given the increasing number of NEOs and their metabolites detected in human urine, such a prioritization is urgently needed to inform targeted public health interventions and regulatory action.

To address the abovementioned research gaps, this study selected representative rural and urban areas in South China, and collected first-morning void urine samples and questionnaires from 184 school-aged children. The major objectives were: (1) to establish a comprehensive quantitative method for determination of both traditional and emerging NEOs in urine samples; (2) to characterize the composition profiles and trace the possible sources of traditional and emerging NEOs in the target population; (3) to estimate the EDI and the corresponding health risks for both single and multiple NEOs; and (4) to develop a ToxPi-based prioritization model that integrates exposure, toxicity, and detection metrics to identify high-priority NEOs requiring control, thereby providing a scientific basis for risk-based chemical management targeting children’s environmental health.

## 2. Materials and Methods

### 2.1. Chemicals and Reagents

Seventeen typical native standards (including 14 parent compounds and 3 metabolites) and their corresponding isotope-labeled internal standards (Table S1) were purchased from Sigma-Aldrich (St. Louis, MO, USA) and Dr. Ehrenstorfer (Augsburg, Germany). MS-grade solvents (methanol, ethyl acetate, glacial acetic acid, and ammonium acetate) were obtained from Merck (Damstadt, Germany), and *β*-glucuronidase/sulfatase was obtained from CNW Technologies (Anpel, Shanghai, China). HPLC-grade water was prepared using a Milli-Q system (Barnstead International, Dubuque, IA, USA).

### 2.2. Study Design and Urine Collection

Typical rural and urban areas in South China were selected for this study, which represent a traditional agricultural region and a populous region, respectively, representing the predominant land use patterns, population density, and agricultural practices typical of South China. Specifically, the rural sites are located in major agricultural production zones, while the urban sites represent residential and commercial districts with minimal direct pesticide application. The distance between two sites is approximately 52 km, and the residents in the two areas have different lifestyle habits and socioeconomic profiles. Considering the operability and accuracy of the first-morning void urine samples collection and the reliability of the questionnaire completed independently, one typical school with the largest student populations in each region were selected, and elementary school students aged 9 or 10 years were randomly recruited, resulting in a total of 184 participants (107 males and 77 females) in 2024. Participants with known diseases, e.g., liver disease, kidney diseases, parathyroid or thyroid disorders, diabetes, cough, etc., were excluded from the present study. Exclusion criteria also included those with unreliable or incomplete questionnaires and those with missing data on sociodemographic characteristics. Following the acquisition of informed consent from all guardians, first-morning void urine samples (40 mL) were collected, aliquoted, and stored at −80 °C until analysis. Additionally, height and body weight were measured, and a questionnaire covering basic information and environmental exposure factors was administered during the survey. This study was approved by the Ethics and Human Subject Committee of Guangdong Provincial Center for Disease Control and Prevention (W96-027E-202419).

### 2.3. Instrumental Analysis

Urine sample pretreatment was conducted using an established protocol, with minor modifications [18,31]. Briefly, 1.0 mL of urine sample was transferred into a 15 mL polypropylene (PP) tube. Then, 1.0 mL of acetic acid-ammonium acetate buffer solution (1.0 mol/L, pH 5.5), 20 μL of *β*-glucuronidase/sulfatase enzyme, and 50 μL of internal standard solution (20 μg/L) were added and mixed. The mixtures were vortex-mixed and incubated overnight at 37 °C. After incubation, 3.0 mL of ethyl acetate was added, and the sample was vigorously shaken for 20 min, followed by centrifugation at 4700 rpm for 10 min. The supernatant was transferred into a fresh 15 mL PP tube. A second extraction was performed with an additional 3 mL of ethyl acetate. The combined extracts were concentrated to near dryness under a gentle nitrogen stream and redissolved in 0.2 mL of 50% methanol-water (*v*/*v*) for instrument analysis.

Seventeen NEOs were identified and quantified by isotope-dilution UPLC-MS/MS (Agilent 1290 UPLC, Sciex Triple Quad 6500+, Santa Clara, CA, USA). Chromatographic separation was performed using a Waters Acquity UPLC BEH C_18_ column (100 mm × 2.1 mm, 1.7 μm) maintained at 35 °C. Analytes were separated using a gradient of mobile phase A (0.5 mM ammonium acetate solution in water) and B (methanol) at a constant flow rate of 0.35 mL/min. The gradient was as follows: 0–0.5 min, 85% A; 0.5–10.0 min, decrease from 85% to 5% A; 10.0–11.5 min, increase from 5% to 85% A; and held for 1.5 min. The total analytical time was 13 min with an injection volume of 5.0 μL.

Quantitative analysis was performed using scheduled multiple reaction monitoring (sMRM) under both positive and negative electrospray ionization modes. The source temperature was 550 °C, with an ionization voltage of −4500 V. The pressures of the nebulizer gas and auxiliary heating gas were both set at 55 psi. The mass spectroscopic parameters are shown in Table S1 and Figure S1.

### 2.4. Quality Assurance and Quality Control

Procedure blanks and laboratory blanks were conducted during the sampling and analyzing procedures. The analytical method was verified by determining the matrix-spike recoveries for each analyte. Randomly selected blank urine was spiked with analyte standards at concentrations of 0.10, 1.0, and 5.0 ng/mL, along with a fixed internal standard concentration (1.0 ng/mL). Each analytical batch included a procedural blank, matrix spikes (1.0 ng/mL), and a random duplicate. All sample concentrations were blank-corrected using the mean value from the procedural blanks. The average recoveries and relative standard deviations for all analytes were determined to be 72.8–108.5% and ≤10.2%, respectively (Table S2). The calibration curves showed excellent linearity (r ≥ 0.9979) over the range of 0.020–20.0 μg/L, validating the method’s reliability for quantification. The relative matrix effects ranged from 83.3% to 106%, which were within the acceptable range.

The method detection limit (MDL) was defined as the minimum measured concentration that could be distinguished from the method blank with 99% confidence [32], and the method quantitation limit (MQL) was set as three times the MDL. The MDLs ranged from 0.01 to 0.08 μg/L, while the MQLs ranged from 0.03 to 0.24 μg/L (Table S1). Negative results were designated as not detected (ND).

### 2.5. Single and Cumulative Exposure and Health Risk Assessment

The health risks associated with both NEOs and m-NEOs were assessed using the relative potency factor (*RPF*) method. Individual NEOs and m-NEOs were integrated into a single metric-imidacloprid equivalent concentration (*IMI*_eq_)—using the toxicity of IMI as a reference value. The *RPF* values for each NEO are listed in Table S3. Since a reference dose (R*f*D) for FLOand SUL was not available, the R*f*D of IMI was applied for these compounds in the current study [24]. Additionally, it was assumed that the R*f*Ds of m-NEOs correspond to those of their respective parent NEOs [7,24]. The *RPF* and IMI_eq_ were calculated as follows [25,33], respectively.

(1) $$RPF_{i}=\frac{RfD_{IMI}}{RfD_{i}}$$

(2) $$\begin{array}{ll} IMI_{eq}\left(NEO_{i}\times RPF_{i}\right) & =IMI\times 1.00+5-OH-IMI\times 1.0+ACE\times 0.80+THM\times 9.50\\ & \;+\;N-dm-ACE\times 0.8+CLO\times 5.82+DIN\times 2.85+THCP\\ & \;\times \;14.25+THCP-AM\times 14.25+NIT\times 3.62+FLO\times 1.00\;+\\ & \;SUL\times 1.00 \end{array}$$

where *RPF*_i_ represents the relative potency factor of the target NEOs or m-NEO, R*f*D_IMI_ (mg/kg/day) signifies the established reference dose for IMI at 0.057 mg/kg/day, and R*f*D_i_ (mg/kg/day) references the established dose of the target NEOs or m-NEO.

The EDI of parent NEOs is generally calculated using Equation (3) [34]. An adjustment of Equation (3) was conducted in order to calculate EDI based on the *RPF* approach by using the U.S. Environmental Protection Agency (EPA) guidelines [35]. To estimate the EDI of parent NEOs based on m-NEO concentrations, Equation (4) was applied [36].

(3) $$EDI_{p}=\frac{C_{p}\times V}{f\times BW}$$

(4) $$EDI_{p}=\frac{C_{m}\times V\times MW_{p}}{f\times BW\times MW_{m}}$$

where *EDI_p_* (μg/kg-bw/day) represents the daily intake of the parent NEO compound through all exposure routes. *C_p_* and *C_m_* (μg/mL) are the concentrations of individual NEOs and their metabolites, respectively. *V* (mL/day) represents the daily urinary excretion rate, which is set to 1.258 L/day for girls and 1.299 L/day for boys [37]. *MW_p_* or *MW_m_* (g/mol) is the molecular weight of the parent NEO or its metabolite. *f* is the excretion proportion, based on human pharmacokinetic data (Table S4). Due to a lack of human pharmacokinetic data, the urine excretion proportions in rats or mice were used instead [38]. For other NEOs with no available human or animal pharmacokinetic data, urine excretion proportions were set to their parent compounds with similar chemical structures [38,39]. BW is the body weight, which varies across countries, gender, and age groups.

To assess the potential non-carcinogenic risk, hazard quotient (HQ) and hazard index (HI) were calculated as follows:

(5) $$HQ=\frac{EDI}{RfD}$$

(6) HI=∑HQ

where HQ (dimensionless) represents the health risk of an individual chemical; HI (dimensionless) indicates the cumulative risk of multiple pollutants [40]; and R*f*D (μg/kg-bw/day) is the reference dose.An HQ or HI greater than 1 indicates a potential non-carcinogenic risk, while values less than 1 indicate negligible risk.

### 2.6. Chemical Prioritization

Five aspects of indices, e.g., concentrations (median value), frequency (DF), toxicity (*RPF*), accumulation (bioconcentration factor, BCF), and persistence (biotransformation half-life), were integrated for chemical prioritization via the Toxicological Prioritization Index (ToxPi, version 2.3). These variables were normalized using the min-max normalization method. The 5 variables were assigned equal weights to ensure that each variable contributed equally and to mitigate human assumption biases [41,42].

### 2.7. StatisticalAnalysis

IBM SPSS version 27.0 (IBM, Armokn, NY, USA) and Origin 2021 (Origin Lab, Northamptn, MA, USA) were used for statistical analyses and creating graphics, respectively. Concentrations below the MDL were replaced by MDL/√2 [43,44]. The normality of NEO concentrations was checked using the *Kolmogorov*–*Smirnov* test. Spearman’s correlation analysis was performed to assess the correlations among NEOs. The *Mann*–*Whitney U* test was conducted to compare NEO exposure between two groups, while the *Kruskal*–*Wallis H* test was used for multiple group comparisons. Multiple linear regression was performed to evaluate the association between urinary concentrations and potential influencing factors (i.e., lifestyle habits and demographic characteristics). Statistical significance was set at *p* < 0.05.

To ensure that the sample size was adequate for robust biomonitoring analysis and risk prioritization, we performed a power analysis using the R package pwr (version 1.30). Based on a medium effect size (Cohen’s f = 0.25, a significance level of α = 0.05, and a desired power (1-β) of 0.80), the estimated minimum sample size was 86 per group. Our final sample size (*n* = 184) exceeded this requirement, confirming sufficient statistical power.

## 3. Results and Discussion

The demographic characteristics and lifestyle habits of the school-aged children are shown in Table 1. All participants were aged 9–10 years, with 58.2% male and 41.8% female.

### 3.1. Concentrations and Composition of Urinary NEOs

As shown in Table 2, among the p-NEOs, CLO and THM were detected in nearly all urine samples, with detection frequencies (DFs) of 98.9% each, followed by ACE (90.2%), FLO (89.1%), IMI (82.6%), SUL (59.8%), NIT (59.2%), and DIN (58.7%). Among the m-NEOs, *N*-dm-ACE showed the highest DF (98.9%), followed by THCP-AM (87.0%) and 5-OH-IMI (77.7%). These results indicate widespread exposure to NEOs among school-aged children, which is consistent with a recent study from Chongqing, Southwest China, reporting DFs ranging from 80.1% to 100.0% [16]. Notably, certain emerging NEOs—including FLO (89.1%), and SUL (59.8%)—exhibited substantially higher DFs than the traditional compounds DIN (58.7%) and THCP (37.0%), suggesting different divergent exposure sources. In the case of THCP-AM, extensive metabolic conversion of its parent compound THCP was identified. This pattern aligns with an earlier survey of elementary school children in South China, in which THCP-AM and THCP were detected in 100% and 20% of urine samples, respectively [45]. NEOs with DF below 50% were excluded from subsequent statistical analysis.

**Figure 1:**
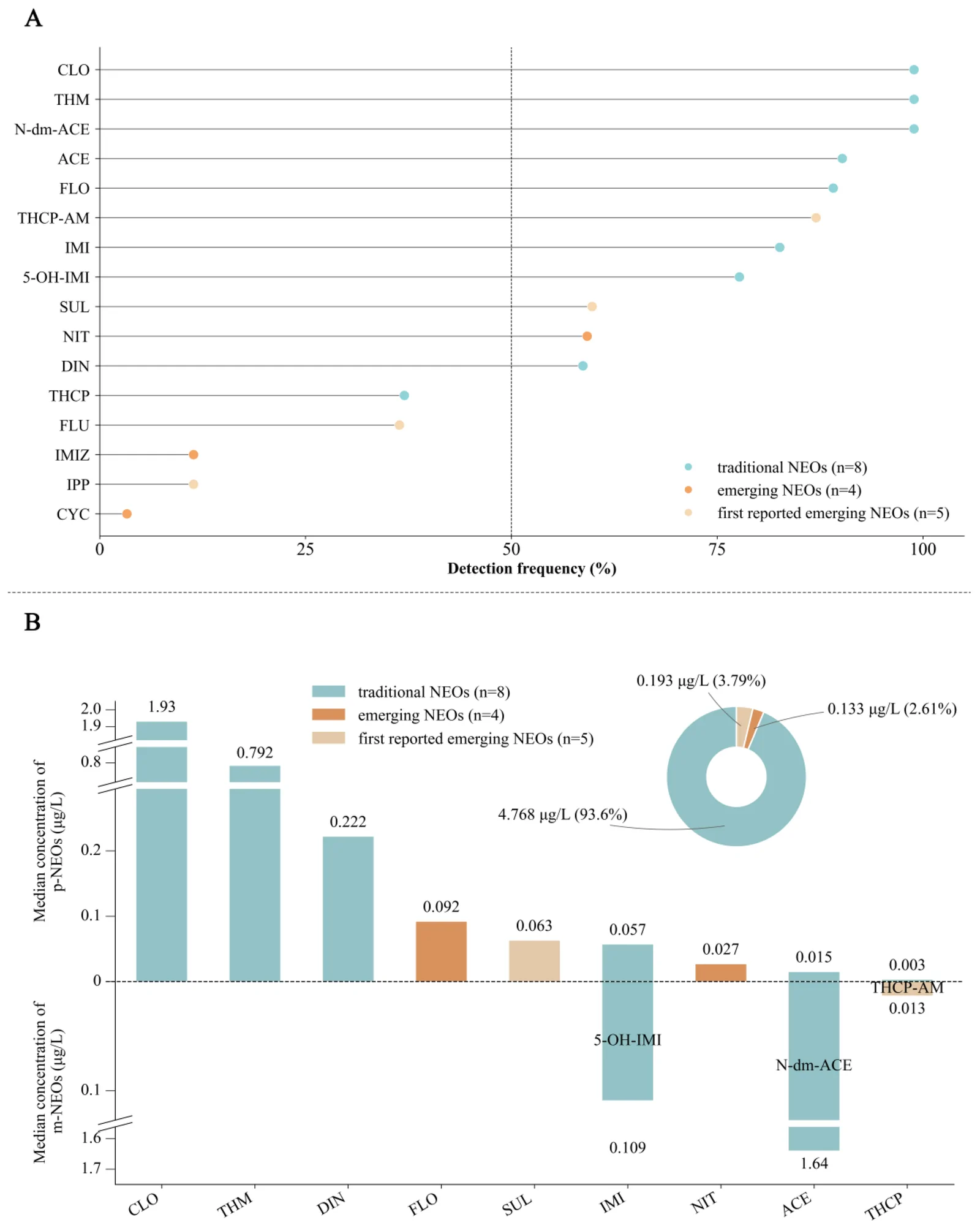
Detection frequencies and median concentrations of traditional and emerging NEOs in school-aged children. (**A**) Detection frequencies of 8 traditional NEOs and 9 emerging NEOs. (**B**) Median concentrations of 8 traditional NEOs and 8 emerging NEOs. The pie represents the cumulative median concentrations of tradition NEOs (n = 8) and emerging NEOs, which are divided into previously reported NEOs (n = 4) and newly identified NEOs (n = 5) in urine samples.

The ∑_12_NEOs ranged from 0.519 to 227 μg/L, with a median value of 8.65 μg/L. This median concentration is approximately 61-fold higher than the median of 0.14 μg/L reported for adults in Shanghai [46]. The five most abundant p-NEOs in our study were CLO (1.93 μg/L), THM (0.792 μg/L), DIN (0.222 μg/L), FLO (0.092 μg/L) and SUL (0.063 μg/L) (Figure 1). The second-generation NEOs CLO and THM presented at significantly higher concentrations than both the first-generation compounds (IMI, ACE, THCP, NIT) and the third-generation counterparts (DIN). This finding aligns with a reported 3.2-fold increase in sales of second-generation NEOs since 2015 [11,24]. Among the m-NEOs, *N*-dm-ACE was the predominant metabolite with a median concentration of 1.64 μg/L, followed by 5-OH-IMI (0.109 μg/L) and THCP-AM (0.013 μg/L). These metabolites originate from the first-generation NEOs, namely ACE, IMI and THCP. The concentrations of m-NEOs were relatively higher than those of the corresponding p-NEOs, which is consistent with previous studies [18,46]. This elevation likely reflects the rapid metabolism of parent NEOs in the body, combined with the higher biological persistence and more efficient urinary excretion of their metabolites.

The median concentrations of CLO (1.93 μg/L) and *N*-dm-ACE (1.64 μg/L) observed in our study were higher than those reported in previous investigations, e.g., children in Q city (CLO: 0.71 μg/L; *N*-dm-ACE: 1.1 μg/L) [4], Shanghai (CLO: <LOD; *N*-dm-ACE: 1.35 μg/L) [40], and the United States (CLO: 0.21 μg/L, *N*-dm-ACE: <LOD) [15]. They were also higher than those documented for pregnant women and the general population from various regions, including China, Japan, and the United States [14,19,47]. This pattern suggests that school-aged children in South China experience relatively higher exposure to CLO and ACE, possibly due to intensive local agricultural use and children’s higher food intake per body weight. In contrast, concentrations of other compounds (DIN, 5-OH-IMI, IMI, and THCP) were consistent with previously reported values (Table S5). The observed inter-study variability may be largely attributable to regional differences in agricultural practices, environmental contamination levels, dietary exposure patterns, and analytical methodologies [25,34].To our knowledge, this is the first study to report several emerging NEOs—namely IPP, SUL, and FLO—in children’s urine samples. Notably, these compounds were detected at measurable concentrations in a subset of the population, with detection frequencies of 11.4%, 68.2%, and 53.4%, respectively. This finding highlights the growing importance of emerging substitutes as contributors to children’s total NEO exposure, a concern that has been largely overlooked in previous biomonitoring studies.

**Figure 2:**
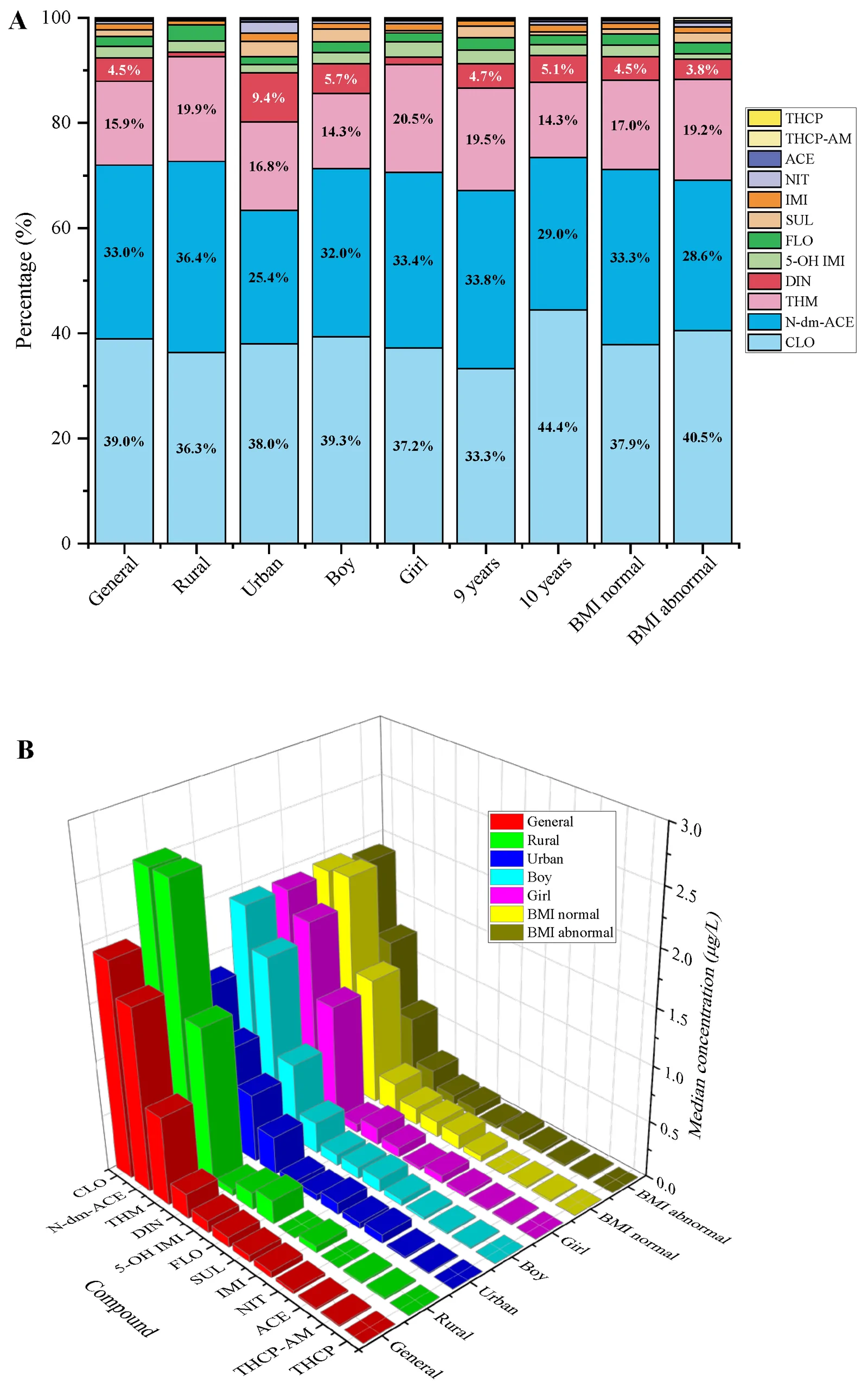
Composition profiles (**A**) and median concentrations (**B**) of NEOs in urine samples.

The composition profiles of urinary NEOs are shown in Figure 2A. CLO, *N*-dm-ACE, THM, and DIN were the predominant contributors, which accounted for 39.0%, 33.0%, 15.9%, and 4.5% of ∑_12_NEOs, respectively. No significant difference was observed by gender. However, a pronounced divergence emerged between rural and urban profiles: *N*-dm-ACE (36.3%) and THM (19.9%) were the dominant contributors in rural school-aged children, whereas their proportions were lower in urban counterparts (25.4% and 16.8%, respectively). CLO remained the predominant component in both groups (approximately 36%). Conversely, DIN accounted for 9.4% of ∑_12_NEOs in urban school-aged children, while they were virtually undetectable in rural children. The urinary concentrations of several NEOs (e.g., CLO, THM, *N*-dm-ACE, DIN, FLO, NIT, THCP-AM and THCP) (Figure 2B) differed significantly between rural and urban participants (*p* < 0.05, Table S6). Specifically, rural children exhibited median levels of CLO, *N*-dm-ACE, THM, 5-OH-IMI, and FLO that were 1.85- to 3.8-fold higher than those in urban children. In contrast, the levels of DIN and SUL were 6.8- and 35-fold lower in rural children compared to their urban counterparts. These distinct exposure patterns between urban and rural populations may be attributed to two non-mutually exclusive factors. First, agricultural application of NEOs is more prevalent in rural areas, whereas non-agricultural sources (including residential biocides and veterinary medicines) are likely more common in urban households. This difference in exposure pathways may explain the higher urinary levels of agricultural-use NEOs (e.g., CLO, THM, *N*-dm-ACE) in rural children, and the higher levels of DIN and SUL—compounds more frequently associated with non-agricultural applications—in their urban counterparts. Second, questionnaire-based surveys suggested distinct dietary patterns. While urban diets rely primarily on produce from external suppliers (e.g., greenhouses in Chinese agricultural zones), rural dietary patterns are more heterogeneous, encompassing both market-sourced and home-grown foods [14]. This divergence in food sources likely leads to differential pesticide exposure, reflecting underlying variations in crop-specific and region-specific pesticide application practices between urban and rural settings [48]. However, direct evidence linking these dietary patterns to specific NEO exposure profiles is currently lacking, in the present study, as we did not collect detailed information on pet ownership, or food frequency. Future studies incorporating such data are warranted to confirm this hypothesis.

**Figure 3:**
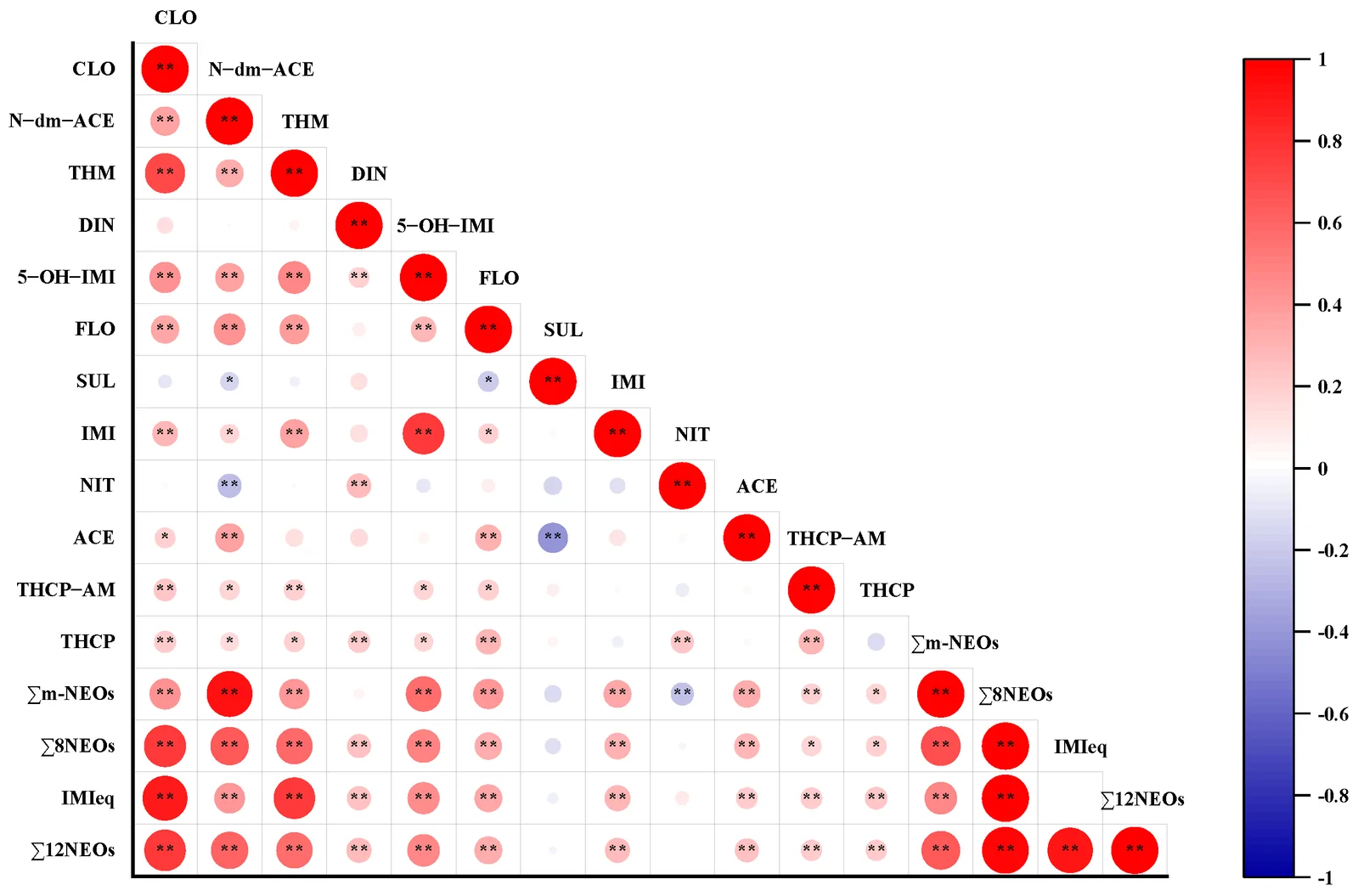
The correlation analysis among concentrations of target compounds. * *p*< 0.05 (two-tailed); ** *p* < 0.01 (two-tailed). Σm-NEOs = the sum concentrations of 3 m-NEOs (5-OH-IMI, *N*-dm-ACE, and THCP-AM); Σ_16_NEOs = the sum concentrations of 16 p-NEOs and 3 m-NEOs; The circle size indicates the magnitude of the correlation coefficient.

Spearman correlation analysis was conducted to examine potential shared exposure sources among the major NEOs (DF > 50%), the sum concentrations of eight traditional NEOs (∑_8_NEOs: IMI, 5-OH-IMI, ACE, *N*-dm-ACE, THCP, THM, DIN, and CLO),∑_12_NEOs, and IMI_eq_ (Figure 3 and Table S7). CLO and THM were strongly correlated (*r* = 0.716, *p* < 0.001). In contrast, THM showed moderate correlations with 5-OH-IMI (*r* = 0.471, *p* < 0.001), IMI (*r* = 0.366, *p* < 0.001), *N*-dm-ACE (*r* = 0.340, *p* < 0.001), and FLO (*r* = 0.387, *p* < 0.01). ∑_8_NEOs strongly correlated with CLO, *N*-dm-ACE, THM, and 5-OH-IMI (r > 0.49, *p* < 0.001), whereas both ∑_12_NEOs and IMI_eq_ showed moderate correlation with ∑_8_NEOs (*r* = 0.908, *p* < 0.001), indicating combined sources from both traditional and emerging NEOs. The significant correlations observed between parent compounds and their metabolites provide evidence of in vivo metabolism. Notably, a strong correlation was found for IMI/5-OH-IMI (*r* = 0.768, *p* < 0.001), whereas weaker but statistically significant correlations were observed for ACE/*N*-dm-ACE (*r* = 0.362, *p* < 0.001) and THCP/THCP-AM (*r* = 0.281, *p* < 0.001). Nevertheless, given the widespread detection of these metabolites in food and water, direct exposure to the metabolites themselves cannot be ruled out as an additional source [12,49,50].

### 3.2. Factors Influencing Urinary NEOs

**Figure 4:**
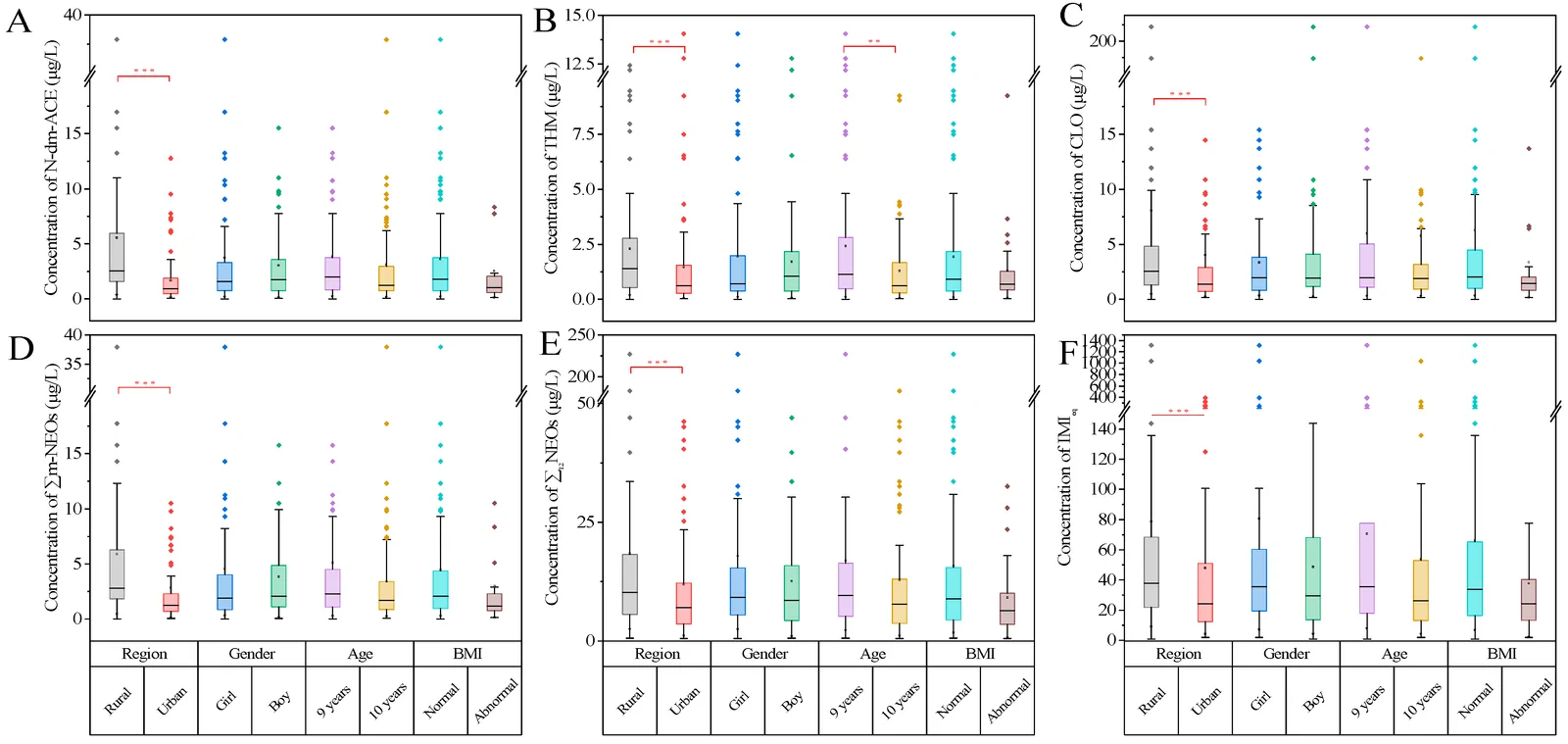
(**A**–**F**) Urinary concentrations of NEOs among school-aged children with different demographic characteristics (*Kruskal*–*Wallis H* and *Mann*–*Whitney U* tests were used). * *p* < 0.05 (two-tailed); ** *p* < 0.01 (two-tailed). Σm-NEOs = the sum concentrations of 3 m-NEOs (5-OH-IMI, *N*-dm-ACE, and THCP-AM); Σ_16_NEOs = the sum concentrations of 13 p-NEOs and 3 m-NEOs. The grouping criteria for the subfigures are provided in Table 1.

As shown in Table S8, significant geographic disparities were observed in urinary NEO profiles. Rural children exhibited significantly higher concentrations of CLO, THM, *N*-dm-ACE, FLO, THCP-AM, ∑m-NEOs, ∑_12_NEOs, and ∑_8_NEOs (all *p* < 0.05, Figure 4), whereas urban children showed elevated levels of NIT, SUL, and DIN (*p* < 0.05, Figure S2). These findings contrast with those of a recent survey of adults, which reported higher urinary NEO concentrations in urban adults (*p* < 0.05) [14]. The rural–urban discrepancy likely reflects crop-specific pesticide application practices and divergent dietary habits between the two areas [14,51].

The urinary concentrations of several NEOs, specifically SUL, ∑_12_NEOs, and ∑_8_NEOs, exhibited statistically significant inverse correlations with family income (*p* < 0.05), suggesting a potential socioeconomic influence on exposure patterns. This inverse relationship contrasts with previous studies reporting positive associations between income and NEO exposure [25]. A plausible explanation for this discrepancy is that the direction of the income–exposure association is route-dependent, distinguishing between scenarios of direct residential use (e.g., home gardening or pest control, more common in higher-income households) and residual dietary intake (the dominant pathway for the general population). This divergence underscores that region-specific socioeconomic conditions are critical determinants of exposure patterns, and that the relationship between income and insecticide exposure may not be universal across different populations and exposure scenarios. The associations between other demographic factors—including age, BMI, gender, and maternal education—and urinary NEO concentrations were also examined. Age was negatively correlated with THM and 5-OH-IMI (*p* < 0.05), while BMI showed positive associations with SUL and THCP-AM (*p* < 0.05). Maternal education was positively correlated with DIN (*p* < 0.05). No other significant relationships were observed (*p* > 0.1). These findings are partially consistent with previous studies. For instance, a BMI–gender interaction was reported for 5-OH-IMI, and *N*-dm-ACE appeared to be inversely associations with obesity-related indicators [15]. Similarly, higher urinary concentrations of CLO, IMI, and IMIZ were reported among girls compared to boys in an urban area of Chongqing, China [16]. In contrast, a study involving pregnant women found that urinary NEO levels were associated with lower education, higher income and higher BMI [25]. These discrepancies between children and pregnant women may reflect differences in study populations, including age-related dietary habits, residential environments, and physiological factors such as metabolic rate.

Beyond demographic characteristics, the potential associations between lifestyle factors—including household insecticide use, agricultural activities, and second-hand smoke exposure—and urinary NEO concentrations were further evaluated. Second-hand smoke exposure showed a positive correlation with THM, IMI, and FLO, consistent with a previous report on pregnant women living in smoking households [39]. No consistent or strong associations were observed for household insecticides (Table S9). Overall, the lifestyle factors examined in this study play a relatively minor role in NEO exposure compared to dietary intake. In contrast, broader demographic characteristics—including age, BMI, urban/rural residence, and socioeconomic status—showed clear patterns. These findings suggest that the determinants of NEO exposure vary substantially across life stages and geographic settings, highlighting the need for population-specific exposure assessment strategies. Furthermore, these demographic and lifestyle determinants provide a contextual basis for interpreting the subsequent ToxPi prioritization of NEOs.

### 3.3. The m-NEO/p-NEORatio as a Biomarker of NEOExposure

The median ratios of m-NEO to their corresponding p-NEOs were 1.85 (5-OH-IMI/IMI), 2.14 (THCP-AM/THCP), and 1034(*N*-dm-ACE/ACE) (Table S10). These results indicated that metabolites were excreted more efficiently in urine than their corresponding parent compounds, or that the parent compounds were extensively metabolized in vivo [18,52]. The exceptionally high ratio for *N*-dm-ACE/ACE suggests that ACE is almost completely converted to its *N*-desmethyl metabolite in humans, a phenomenon that warrants further toxicological attention.

The m-NEO/p-NEO ratios were significantly associated with specific demographic and lifestyle factors (*p* < 0.05, Table S11). The 5-OH-IMI/IMI ratio showed a statistically significant but weak negative correlation with age (*r* = −0.224, *p* = 0.002). Rural participants exhibited higher ratios of 5-OH-IMI/IMI (2.24 vs. 1.33, *p*< 0.001) than urban counterparts (Tables S8 and S11). Sex and BMI also influenced the THCP-AM/THCP ratio: it was lower in boys than girls (1.77 vs. 2.91, *p* = 0.038) and lower in individuals with abnormal BMI than normal BMI (1.82 vs. 4.11, *p* = 0.005). These differences, though statistically significant, had small effect sizes (r: 0.15–0.21). *N*-dm-ACE, 5-OH-IMI, and THCP-AM were recognized as metabolic products of NEOs in animals. Our findings provide critical human biomonitoring evidence supporting this pathway. To the best of our knowledge, this is the first report of gender- and BMI-related differences in the ratio of THCP-AM/THCP in humans. Collectively, the m-NEO/p-NEO ratios may serve as useful biomarkers for assessing human exposure to NEOs [18].

### 3.4. Health Risk Assessment and ToxPi-Based Prioritization of NEOs

**Figure 5:**
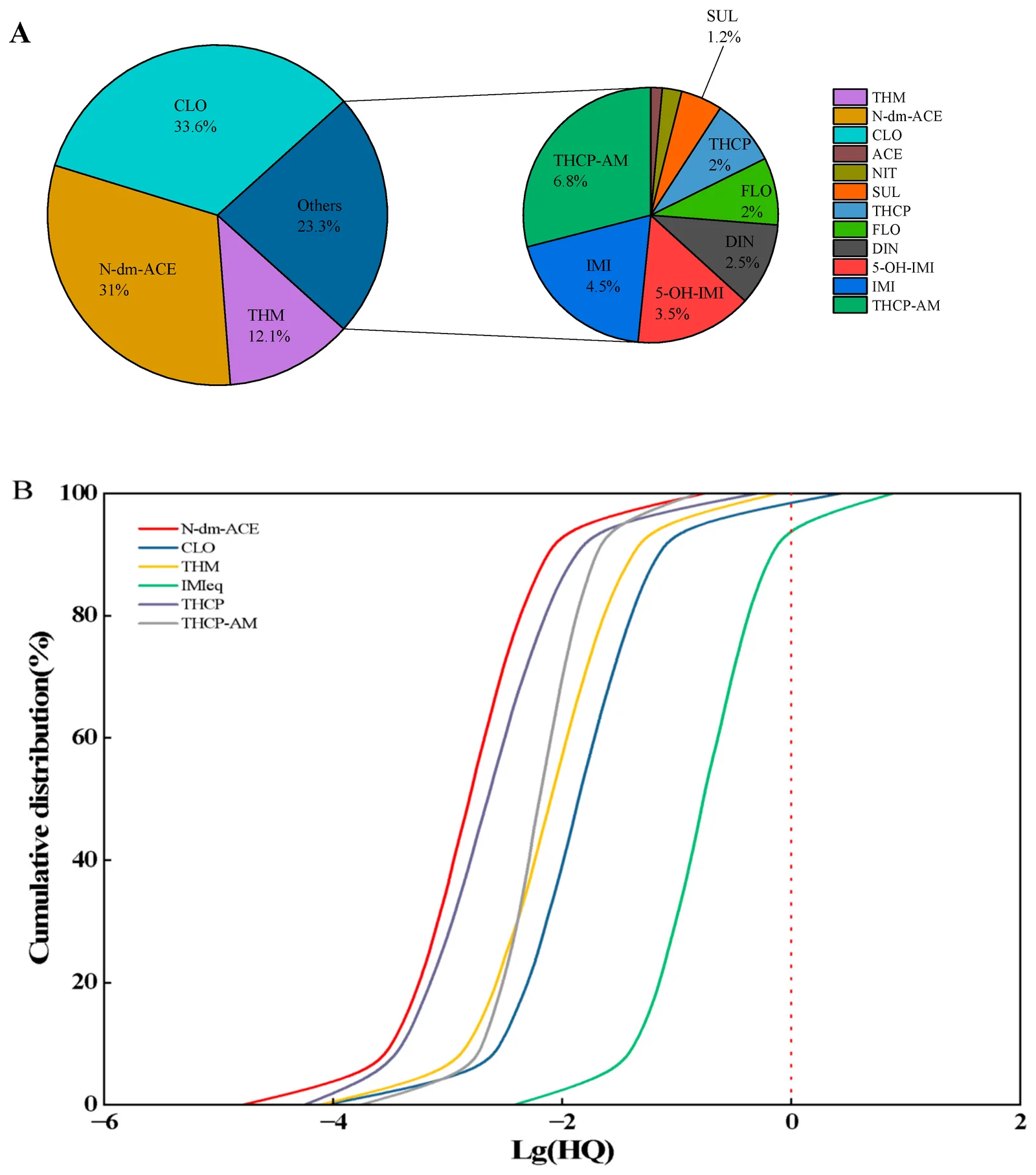
The contributions of median EDI values of NEOs in urine (**A**) and *Monte Carlo* simulation of NEOs exposure based on R*f*D (**B**). The red dotted line represents the baseline (zero line), corresponding to a value of 0 on the Y-axis.

The EDI values of NEOs based on urinary concentrations are summarized in Table 3. Among the individual compounds, CLO exhibited the highest median EDI (0.127 μg/kg-bw/day), accounting for 33.6% of the total NEO burden, followed by *N*-dm-ACE (0.117 μg/kg-bw/day, 31.0%), THM (0.046 μg/kg-bw/day, 12.1%), and THCP-AM (0.026 μg/kg-bw/day, 6.8%) (Figure 5A). The EDI value of CLO in our study (0.127 μg/kg-bw/day) exceeded those previously reported values for the general population in six Chinese cities (0.050 μg/kg-bw/day) [14], young adults in Guangdong (0.038 μg/kg-bw/day) [53], and middle school students in Chongqing (0.0175 μg/kg-bw/day) [54] (Table S12). Similarly, the EDI value of *N*-dm-ACE (0.117 μg/kg-bw/day) was 1.82-fold higher than that reported for pregnant women in Wuhan (0.064 μg/kg-bw/day) [55], and 1.73-to 3.45-fold higher than those observed among young adults in Guangdong [56]. Moreover, THCP-AM had a substantially higher EDI value than the other NEOs. In contrast, the median EDIs of THM, DIN, and 5-OH-IMI were lower than those reported for general population in six Chinese cities, suggesting a distinct exposure profile [14,56]. 

The median EDI of ∑_12_NEOs (0.696 μg/kg-bw/day) was 3.38-fold higher than that reported for young adults in Guangdong (0.206 μg/kg-bw/day) [53], and substantially higher than those observed in other populations [25] (Table S12). Consistent with previous reports that children exhibited higher EDI values for NEOs and their metabolites than adults due to dietary exposure [24], our results support this finding. The IMI_eq_ value (2.83 μg/kg-bw/day) was comparable to that reported for six cities in China (3.31 μg/kg-bw/day) and much higher than those documented in other Chinese and Japanese populations [25]. Although exposure levels for individual NEOs were relatively low, the elevated EDI for co-exposure highlights the importance of mixture toxicity.

The median HQ values were 0.013for CLO, 0.0076 for THM, 0.0064 for THCP-AM, and 0.0019 for THCP (Table S13). At the 95th percentile, the HQ values increased to 0.0825 (CLO), 0.070 (THM), 0.040 (THCP), and 0.024 (THCP-AM). For single compounds, only CLO had a small proportion (0.54%) of HQ exceeding 1.0 (Figure 5B). At the same time, 1.09% of participants exhibited HQs exceeding the safety threshold of 1 for both IMI_eq_ and ∑_16_NEOs, indicating potential health risks from elevated NEO exposure. This pattern is consistent with the intensive agricultural use of NEOs in Guangdong Province [11].

**Figure 6:**
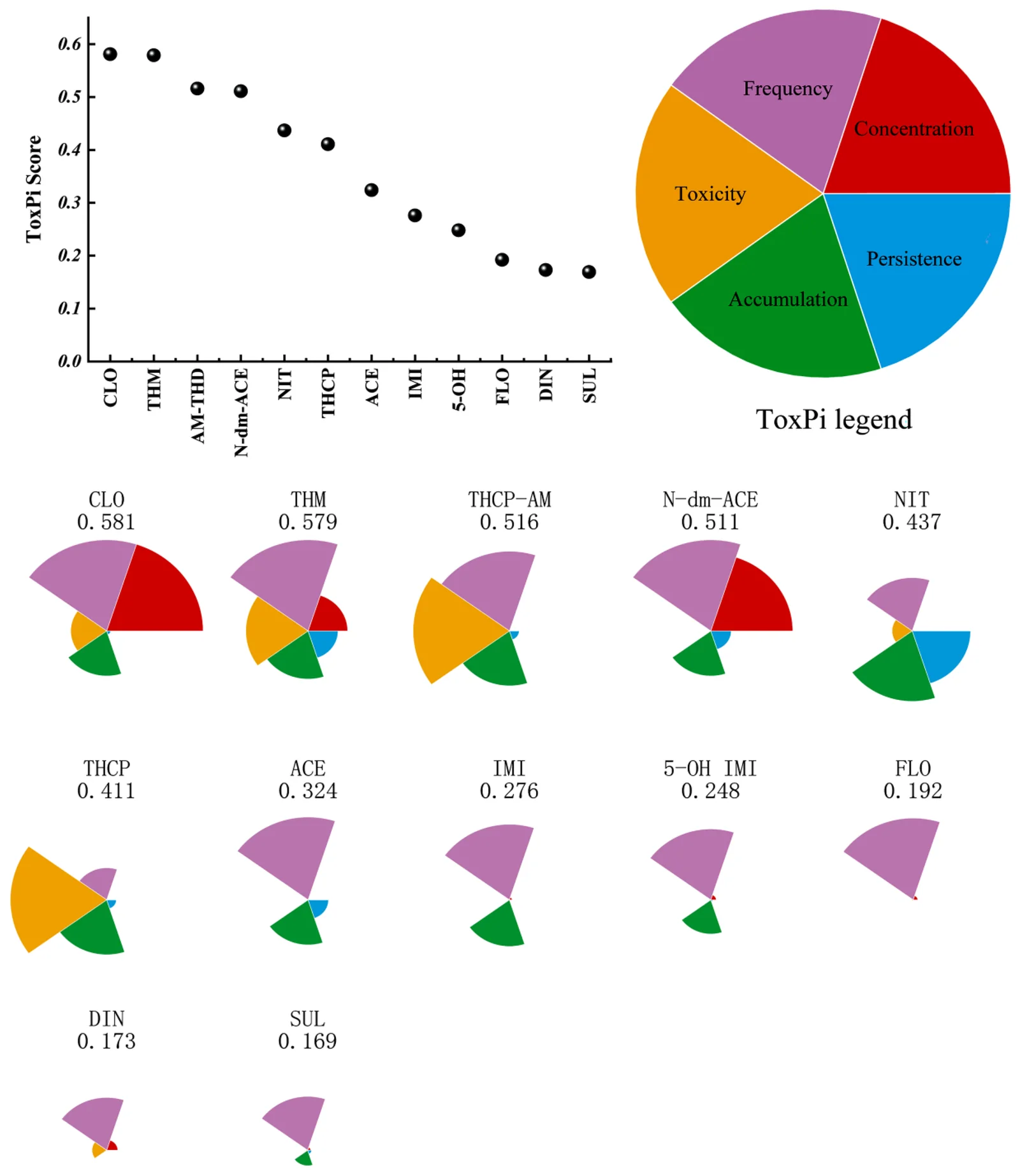
Ranking of NEOs based on the ToxPi score, which consist of concentrations (red), frequancy (DF, purple), toxicity (*RPF*, yellow), accumulation (BCF, green), persistence (half-life, blue).

To facilitate the identification of priority of NEOs for children’s health protection, we applied the ToxPi model. This framework integrates disparate data streams into transparent, visual rankings. Our Toxpi model incorporated five key dimensions: urinary concentrations, frequency, toxicity, bioaccumulation potential, and persistence (Table S14). All criteria were assigned equal weight to avoid subjective bias. The specific scores and contributions are illustrated in Figure 6. The top-four ToxPi scores were CLO (0.581), THM (0.579), THCP-AM (0.516), and *N*-dm-ACE (0.511), respectively. Their high rankings were primarily driven by high exposure metrics (CLO and *N*-dm-ACE via high concentrations; all withhigh DFs > 85%) and elevated toxicity (THCP-AM and THM). By contrast, NIT achieved moderately high ToxPi scores not from exposure magnitude, but from itsrelatively greater persistence and bioaccumulation potential, underscoring the value of a multi-domain assessment: compounds may rank high due to different hazard exposure fate combinations, each carrying different implications for risk management. The high-priority status of CLO, THM, THCP-AM, and *N*-dm-ACE is reinforced by their frequent detection in surface waters, groundwater, drinking water, and wastewater in China [12,42]. Given the high water solubility of NEOs and the particular vulnerability of children, we propose that these four compounds be considered priority chemicals for regulatory attention in child-related environmental health policies. Establishing environmental quality standards for them would provide a science-based foundation for mitigating children’s exposure through water and food pathways.

### 3.5. Strengths and Limitations

This study has several strengths. First, to our knowledge, it provides the most comprehensive profiling of both traditional and emerging NEO exposures in school-aged children. This comprehensive analytical approach enabled us to characterize exposure levels, identify potential sources, and elucidate exposure pathways in this vulnerable population. Second, by applying the ToxPi model, we translated complex biomonitoring data into a transparent, policy relevant prioritization of NEOs. This represents the first application of ToxPi to prioritize NEOs in a human biomonitoring context, providing a science-based list of priority compounds (CLO, THM, THCP-AM, and *N*-dm-ACE) for regulatory attention in children’s environmental health. Third, we identified gender- and BMI-related differences in m-NEO/p-NEO ratios, which may serve as useful exposure biomarkers. Four, we evaluated the associated health risks, contributing to the growing body of knowledge regarding exposure to and risks of emerging NEO substitutes.

Nevertheless, several limitations should be acknowledged. First, the use of first-morning urine samples may not accurately reflect medium- or long-term exposure, given the relatively short half-lives (hours to days) of most NEOs and the generally low intra-class correlation coefficients reported for their urinary metabolites. Second, the lack of urinary creatinine or specific gravity adjustment may have influenced the precision of our exposure predictor analysis, and future studies should incorporate such measurements. Third, the absence of detailed dietary records and information on household insecticide use limits a more precise characterization of exposure sources. Fourth, the lack of human pharmacokinetic data forced us to rely on animal-derived pharmacokinetic data for certain NEOs (e.g., CYC, THCP, NIT, THM), introducing uncertainty into exposure estimates. At the same time, since no human or animal pharmacokinetic data were available for certain NEOs (e.g., FLO), their urine excretion proportions were set to those of compounds with a similar chemical structure of the parent compound, which may further increase the uncertainty. Fifth, while we adopted the MDL/√2 substitution method for left-censored data, our primary inferences were based on median concentrations, which are robust to such substitution. Nonetheless, this approach may underestimate variance, and caution is warranted when interpreting absolute mean values and percentiles near the MDL.

## 4. Conclusions

This study reveals the widespread exposure to both traditional and emerging NEOs among school-aged children in South China. CLO, THM, and *N*-dm-ACE were identified as the predominant compounds. To our knowledge, this is the first report of urinary detection of several emerging NEOs (SUL, FLO, and IPP) in this population. The ratio of metabolites to parent compounds (e.g., *N*-dm-ACE/ACE, 5-OH-IMI/IMI, THCP-AM/THCP) was identified as a useful biomarker for assessing human exposure to NEOs, with gender- and BMI-related differences observed for THCP-AM/THCP. While the HQs for individual NEOs remained below the safety threshold for most children, cumulative exposure assessment revealed a notably elevated EDI under co-exposure scenarios. The school-aged children in our study generally exhibited higher EDI values than those reported for pregnant women or adults, underscoring their increased vulnerability. Using the ToxPi model, CLO, THM, THCP-AM, and *N*-dm-ACE were identified as high-priority compounds requiring regulatory attention for children’s environmental health. These results underscore the critical need for continued biomonitoring and risk assessment of NEOs in children, particularly for emerging substitutes and their metabolites that are not adequately covered by current safety guidelines. Future studies should prioritize mixture toxicity evaluation and incorporate longitudinal designs to better characterize realistic health implications of co-exposure in vulnerable populations.

## Figures and Tables

**Table 1:** Demographic characteristics of school-aged children (n = 184).

		Number	Proportion (%)			Number	Proportion (%)
Regions				Family income (yuan/month)			
	Urban	99	53.8		<1500	35	19.0
	Rural	85	46.2		1500–2999	36	19.6
Age (years)					3000–4999	74	40.2
	9	91	49.5		≥5000	39	21.2
	10	93	50.5	Maternal educational attainment			
Gender					Primary school and below	17	9.2
	Male	107	58.2		Middle school	86	46.7
	Female	77	41.8		Senior High School	37	20.1
BMI ^a^					Diploma and bachelor’s degree	44	23.9
Male							
	≤19.8	95	88.8				
	>19.8	12	11.2				
Female							
	≤19.0	64	83.1				
	>19.0	13	16.9				

^a^ Classified by overweight as 19.8 for male and 19.0 for female at the age of 9.5 (Chinese hygiene standards WS/T 586—2018, screening for overweight and obesity among school-aged children and adolescents).

**Table 2:** Concentrations of NEOs in urine samples among school-aged children (n = 184, μg/L).

	Median	DF(%) *	Mean ± SD	GM	Range	P95
CLO	1.93	98.9	5.89 ± 21.3	1.94	<MDL–222	13.7
*N*-dm-ACE	1.64	98.9	3.47 ± 7.71	1.52	<MDL–89.7	10.4
THM	0.792	98.9	1.85 ± 2.60	0.838	<MDL–14.1	7.97
DIN	0.222	58.7	1.73 ± 4.72	0.087	<MDL–38.6	10.3
5-OH-IMI	0.109	77.7	0.768 ± 4.35	0.075	<MDL–51.3	1.91
FLO	0.092	89.1	0.247 ± 0.382	0.067	<MDL–2.49	0.893
SUL	0.063	59.8	0.564 ± 1.96	0.047	<MDL–20.6	1.80
IMI	0.057	82.6	0.213 ± 0.906	0.050	<MDL–11.3	0.615
NIT	0.027	59.2	0.114 ± 0.269	0.023	<MDL–3.00	0.456
ACE	0.015	90.2	0.030 ± 0.069	0.014	<MDL–0.661	0.08
THCP-AM	0.013	87.0	0.016 ± 0.014	0.012	<MDL–0.075	0.047
THCP	0.003	51.0	<MQL	<MQL	<MDL–0.231	<MQL
IPP	<MQL	11.4	<MQL	<MQL	<MDL–4.46	<MQL
IMIZ	<MQL	11.4	<MQL	<MQL	<MDL–2.39	<MQL
CYC	<MQL	3.30	<MQL	<MQL	<MDL–13.8	<MQL
FLU	<MQL	36.4	<MQL	<MQL	<MDL–0.131	<MQL
∑m-NEOs	4.29		10.1 ± 23.5	4.52	0.007–224	31.4
∑_8_NEOs	7.84		14.0 ± 25.0	7.34	0.02–227	39.6
IMIeq	31.7		62.2 ± 131	31.4	0.736–1314	188
∑_12_NEOs	8.65		14.9 ± 25.2	8.46	0.519–227	42.3

* DF = detection frequency; GM = geometric mean; Σm-NEOs = the sum concentrations of 3 m-NEOs (5-OH-IMI, N-dm-ACE, and THCP-AM); Σ_8_NEOs = the sum concentrations of 8 traditional NEOs (IMI, 5-OH-IMI, ACE, N-dm-ACE, THCP, THM, DIN, and CLO); Σ_12_NEOs = the sum concentrations of 9 p-NEOs and 3 m-NEOs. Values below MQL were imputed as MQL/√2 for calculation of means and medians; P95 was estimated using the Kaplan–Meier method; Mean±SD are presented for descriptive purposes only; all statistical comparisons were based on median concentrations; For analytes with detection frequency < 50%, all quantitative summary statistics (Median, Mean ± SD, GM, and P95) have been replaced with “< MQL”, as the quantitative summary statistics are not reported due to the high proportion of censored data; Detection frequencies are provided for each analyte to inform interpretation.

**Table 3:** Estimated daily intake (EDI, μg/kg-bw/day) of NEOs exposure in urine.

	Median	GM	Mean	Min	Max	P25	P75	P95
CLO	1.27 × 10^−1^	1.29 × 10^−1^	4.13 × 10^−1^	5.80 × 10^−5^	1.80 × 10^1^	6.42 × 10^−2^	2.83 × 10^−1^	8.25 × 10^−1^
N-dm-ACE	1.17 × 10^−1^	1.12 × 10^−1^	2.63 × 10^−1^	1.89 × 10^−4^	7.69	5.36 × 10^−2^	2.70 × 10^−1^	7.92 × 10^−1^
THM	4.58 × 10^−2^	4.44 × 10^−2^	1.00 × 10^−1^	1.38 × 10^−4^	1.01	1.92 × 10^−2^	1.08 × 10^−1^	4.18 × 10^−1^
THCP-AM	2.56 × 10^−2^	2.50 × 10^−2^	3.49 × 10^−2^	5.10 × 10^−3^	1.83 × 10^−1^	1.42 × 10^−2^	4.56 × 10^−2^	9.63 × 10^−2^
IMI	1.71 × 10^−2^	1.48 × 10^−2^	6.46 × 10^−2^	5.44 × 10^−4^	3.68	6.45 × 10^−3^	4.85 × 10^−2^	1.56 × 10^−1^
5-OH-IMI	1.31 × 10^−2^	9.07 × 10^−3^	9.36 × 10^−2^	2.15 × 10^−4^	6.74	2.00 × 10^−3^	3.69 × 10^−2^	2.00 × 10^−1^
DIN	9.27 × 10^−3^	3.85 × 10^−3^	8.03 × 10^−2^	7.24 × 10^−5^	1.57	1.44 × 10^−4^	4.52 × 10^−2^	5.14 × 10^−1^
FLO	7.54 × 10^−3^	5.23 × 10^−3^	2.00 × 10^−2^	4.63 × 10^−5^	2.36 × 10^−1^	1.55 × 10^−3^	2.36 × 10^−2^	7.36 × 10^−2^
THCP	7.50 × 10^−3^	1.24 × 10^−2^	3.23 × 10^−2^	3.52 × 10^−3^	5.31 × 10^−1^	6.02 × 10^−3^	2.35 × 10^−2^	1.59 × 10^−1^
SUL	4.65 × 10^−3^	3.69 × 10^−3^	4.53 × 10^−2^	1.35 × 10^−4^	1.69	2.65 × 10^−4^	3.68 × 10^−2^	1.17 × 10^−1^
NIT	2.19 × 10^−3^	2.00 × 10^−3^	1.04 × 10^−2^	1.48 × 10^−4^	2.35 × 10^−1^	2.73 × 10^−4^	1.10 × 10^−2^	4.80 × 10^−2^
ACE	1.27 × 10^−3^	1.19 × 10^−3^	2.58 × 10^−3^	4.92 × 10^−5^	5.11 × 10^−2^	8.29 × 10^−4^	1.93 × 10^−3^	7.02 × 10^−3^
∑m-NEOs	4.19 × 10^−1^	4.29 × 10^−1^	8.10 × 10^−1^	3.99 × 10^−2^	1.82 × 10	2.25 × 10^−1^	7.87 × 10^−1^	2.30
∑_8_NEOs	5.80 × 10^−1^	5.43 × 10^−1^	1.05 × 10^0^	7.27 × 10^−3^	1.83 × 10	2.77 × 10^−1^	1.05	2.66
IMIeq	2.83	2.90	5.06	2.07 × 10^−1^	1.06 × 10^2^	1.51	4.99	1.57 × 10
∑_12_NEOs	6.96 × 10^−1^	7.08 × 10^−1^	1.20	5.24 × 10^−2^	1.84 × 10	3.76 × 10^−1^	1.20	3.01

GM = geometric mean; Σm-NEOs = the sum concentrations of 3 m-NEOs (5-OH-IMI, N-dm-ACE, and THCP-AM); Σ_8_NEOs = the suml concentrations of 8 traditional NEOs (IMI, 5-OH-IMI, ACE, N-dm-ACE, THCP, THM, DIN, and CLO); Σ_12_NEOs = the sum concentrations of 9 p-NEOs and 3 m-NEOs.

## Data Availability

The original contributions presented in this study are included in the article/Supplementary Material. Further inquiries can be directed to the corresponding authors.

## Supplementary Materials

The following supporting information can be downloaded at: https://www.mdpi.com/article/10.3390/toxics14080735/s1, Figure S1: The chromatography of 16 NEOs in standard solution (10 μg/L); Figure S2: Urinary concentrations of NEOs among school-aged children between rural and urban areas. Table S1: MRM Transitions and Other LC-MS/MS Conditions; Table S2: Recoveries and precision of NEOs in urine samples of children (n = 8); Table S3: Reference dose (R*f*D) and relative potency factors (*RPF*) for NEOs; Table S4:Excretion proportion values(*f*) of NEOs in urine; Table S5: Summary of mean (median) concentrations (μg/L) of NEOs in urine samples from this study with previous studies; Table S6: Region-specific levels of NEOs in urine samples (μg/L); Table S7: Spearman’s correlation coefficient of NEOs; Table S8: Concentrations of NEOs with different demographic characteristics; Table S9: Concentrations of NEOs in urine samples among school-aged children with different lifestyle habits; Table S10: Concentration ratios of m-NEOs/p-NEOs in urine samples; Table S11: Effects of demographic characteristics on ratio of m-NEO/p-NEO; Table S12: Summary of median EDI (μg/kg bw/day) of NEOs from this study with previous studies; Table S13: Non-carcinogenic risk (HQ) of NEO exposure in urine samples among school-aged children; Table S14: Concentrations, frequancy, toxicity, accumulation, persistence and ToxPi scores of NEOs. References [14,15,16,18,19,20,21,24,25,26,31,38,39,40,45,46,47,49,50,52,53,54,55,56,57,58,59,60,61,62,63,64] are cited in Supplementary Materials.

## Abbreviations

The following abbreviations are used in this manuscript:

NEOs	neonicotinoids
∑_12_NEOs	sum concentrations of 12 NEOs
CLO	clothianidin
*N*-dm-ACE	*N*-desmethyl-acetamiprid
THM	thiamethoxam
DIN	dinotefuran
IPP	paichongding
SUL	sulfoxaflor
FLO	flonicamid
EDI	estimated daily intake
ToxPi	Toxicological Priority Index
THCP-AM	thiacloprid-amid
IMI	imidacloprid
ACE	acetamiprid
THCP	thiacloprid
NIT	nitenpyram
5-OH-IMI	5-hydroxy-imidacloprid
CYC	cycloxaprid
IMIZ	imidaclothiz
MDL	method detection limit
MQL	method quantitation limit
ND	not detected
*RPF*	relative potency factor
IMI_eq_	metric-imidacloprid equivalent concentration
R*f*D	reference dose
p-NEO	parent NEO
m-NEO	NEO metabolite
HQ	hazard quotient
HI	hazard index
DF	detection frequency
