# Supplementary material for: Urinary Biomonitoring and Risk Prioritization of Traditional and Emerging Neonicotinoids in School-Aged Children, South China
Source: Toxics. 2026 Aug 21;14(8):735. doi: 10.3390/toxics14080735 (PMC13517688; doi:10.3390/toxics14080735)
Supplement: Supplementary file 1 [file toxics-14-00735-s001.zip › toxics-4453602-supplementary.pdf]

Supplemental material

Urinary biomonitoring and risk prioritization of traditional and emerging neonicotinoids in school-aged children, South China

Pan Zhu[a,#], Ling-Chuan Guo[b,#], Xuan Liu [a,c], Junwei Yang[a], Guangning Su[a], Jing Deng[a], Xiuhua Zhong[a], Chaoyang Long[a], Jingguang Li[d,*], Shengbing Yu[a,*]

[a] *Guangdong Provincial Center for Disease Control and Prevention, Guangzhou, 511430, Guangdong, China*

b State Key Laboratory of Environmental Criteria and Risk Assessment, State Environmental Protection Key Laboratory of Ecological Effect and Risk Assessment of Chemicals, Chinese Research Academy of Environmental Sciences, Beijing 100012, China

c Guangdong-Hong Kong-Macao Joint Laboratory for Contaminants Exposure and Health, Guangdong Key Laboratory of Environmental Catalysis and Health Risk Control, Institute of Environmental Health and Pollution Control, Guangdong University of Technology, Guangzhou, 510006, Guangdong, China

d China National Center for Food Safety Risk Assessment, Beijing, 100012, China.

* Corresponding Author: Jingguang Li, E-mail: lijg@cfsa.net.cn; Shengbing Yu;,E-mail: shengbingyu@sina.com.

# These authors contributed equally to the work.

List of contents

Figures:

Figure S1. The chromatography of 16 NEOs in standard solution (10 μg/L).

Figure S2. Urinary concentrations of NEOs among school-aged children between rural and urban areas.

Tables:

Table S1 MRM Transitions and Other LC-MS/MS Conditions.

Table S2 Recoveries and precision of NEOs in urine samples of children (n=8).

Table S3 Reference dose (R*f*D) and relative potency factors (RPF) for NEOs.

Table S4 Excretion proportion values(*f*) of NEOs in urine.

Table S5 Summary of mean (median) concentrations (μg/L) of NEOs in urine samples from this study with previous studies.

Table S6 Region-specific levels of NEOs in urine samples (μg/L).

Table S7 Spearman's correlation coefficient of NEOs

Table S8 Concentrations of NEOs with different demographic characteristics.

Table S9 Concentrations of NEOs in urine samples among school-aged children with different lifestyle habits.

Table S10 Concentration ratios of m-NEOs/p-NEOs in urine samples.

Table S11 Effects of demographic characteristics on ratio of m-NEO/p-NEO.

Table S12 Summary of median EDI (μg/kg bw/day) of NEOs from this study with previous studies.

Table S13 Non-carcinogenic risk (HQ) of NEO exposure in urine samples among school-aged children.

Table S14 Concentrations, frequancy, toxicity, accumulation, persistence and ToxPi scores of NEOs.

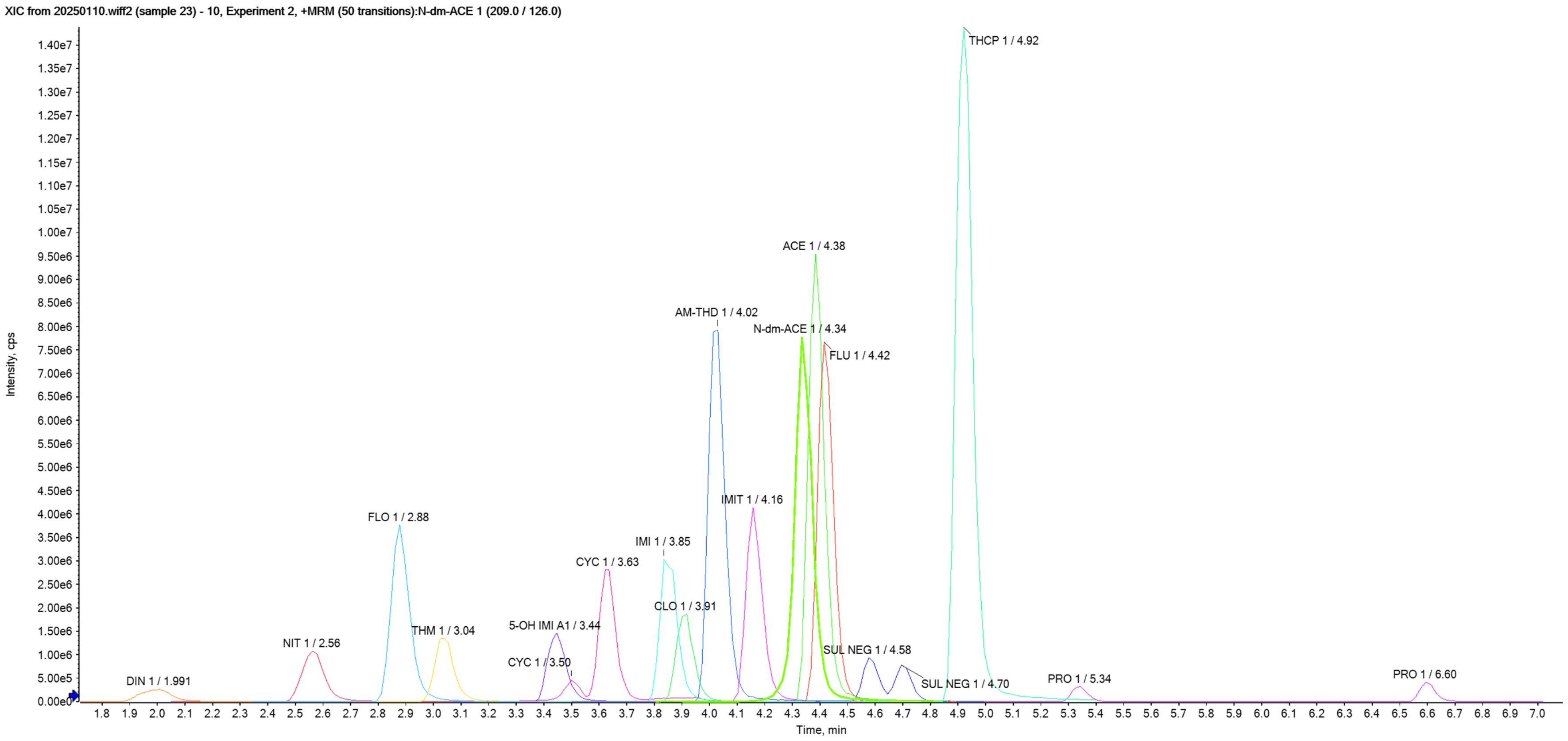

Figure S1. Representative chromatographic separation of 16 NEOs in standard solution (10 μg/L).

(* Paichongding (IPP) exhibits two chromatographic peaks, likely attributable to geometric isomerism (cis/trans), consistent with the structural characteristics of its core.)

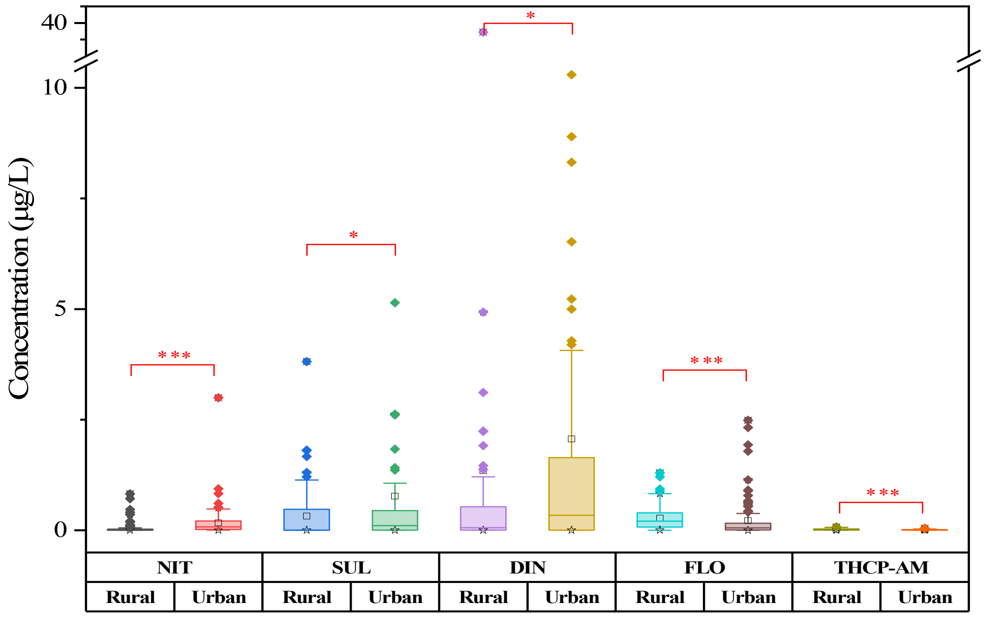

Figure S2. Urinary concentrations of NEOs among school-aged children between rural and urban areas. (*Kruskal–Wallis H* and *Mann–Whitney U* tests were used. $^{*}p < 0.05$ (two-tailed); $^{**}p < 0.01$ (two-tailed).

Table S1 MRM Transitions and Other LC-MS/MS Conditions.

**Analytes and internal standards**	**Abbrev.**	**CAS No.**	**Formula**	**Mode**	**Parent m/z**	**Product ① m/z**	**Product ② m/z**	**DP (V)**	**CE (eV)**	**MDL (μg/L)**	**MQL (μg/L)**
Paichongding	IPP	948994-16-9	$C_{17}H_{23}ClN_4O_3$	positive	367	263	306	80	22, 35	0.02	0.06
Imidaclothiz	IMIZ	105843-36-5	$C_7H_8ClN_5O_2S$	positive	262	181	122	30	19, 37	0.08	0.24
Cycloxaprid	CYC	1203791-41-6	$C_{14}H_{15}ClN_4O_3$	positive	323	126	276	70	45, 25	0.05	0.15
Nitenpyram	NIT	150824-47-8	$C_{11}H_{15}ClN_4O_2$	positive	271	126	224	30	33, 19	0.03	0.09
Flupyradifurone	FLU	951659-40-8	$C_{12}H_{11}ClF_2N_2O_2$	positive	289	126	90	70	27, 59	0.01	0.05
Sulfoxaflor	SUL	946578-00-3	$C_{10}H_{10}F_3N_3OS$	negative	276	231	212	-30	-22, -20	0.03	0.09
Dinotefuran	DIN	165252-70-0	$C_7H_{14}N_4O_3$	positive	203	87	73	25	20, 26	0.02	0.06
Thiamethoxam	THM	153719-23-4	$C_8H_{10}ClN_5O_3S$	positive	292	181	132	30	30, 25	0.02	0.06
Acetamiprid	ACE	135410-20-7	$C_{10}H_{11}ClN_4$	positive	223	126	56	55	30, 18	0.02	0.06
Clothianidin	CLO	210880-92-5	$C_6H_8ClN_5O_2S$	positive	250	169	132.1	30	16, 20	0.01	0.03
Thiacloprid	THCP	111988-49-9	$C_{10}H_9ClN_4S$	positive	253	126	99	60	27, 55	0.02	0.06
Imidacloprid	IMI	138261-41-3	$C_9H_{10}ClN_5O_2$	positive	256	209	175	40	20, 26	0.02	0.06
Flonicamid	FLO	158062-67-0	$C_9H_6F_3N_3O$	positive	230	203	148	80	22, 35	0.01	0.03
N-desmethyl-acetamiprid	N-dm-ACE	190604-92-3	$C_9H_9ClN_4$	positive	209	126	90	50	25, 43	0.01	0.03
5-hydroxy imidacloprid	5-OH-IMI	155802-61-2	$C_9H_{10}ClN_5O_3$	positive	272	225	191	30	20, 23	0.02	0.06
Thiacloprid-Amid	THCP-AM	676228-91-4	$C_{10}H_{11}ClN_4OS$	positive	271	126	90	30	38, 61	0.02	0.06
Imidaclothiz-d_4	IMIZ-d_4	105843-36-5	$C_7H_4D_4ClN_5O_2S$	positive	266	185	/	30	22	/	
Nitenpyram-d_3	NIT-d_3	150824-47-8	$C_{11}H_{12}D_3ClN_4OS$	positive	274	228	/	40	18	/	
Sulfoxaflor-d_3	SUL-d_3	946578-00-3	$C_{10}H_7D_3F_3N_3OS$	negative	279	214	/	-30	-22	/	
Dinotefuran-d_3	DIN-d_3	/	$C_7H_{11}D_3N_4O_3$	positive	206	132	/	20	19	/	

Thiamethoxam-d4	THM-d3	1331642-98-8	$C_8H_6D_4ClN_5O_3S$	positive	295	184	/	30	30	/
Acetamiprid-d3	ACE-d3	1353869-35-8	$C_{10}H_8D_3ClN_4$	positive	226	126	/	45	30	/
Clothianidin-d3	CLO-d3	1262776-24-8	$C_6H_5D_3ClN_5O_2S$	positive	253	132	/	35	20	/
Thiacloprid-d4	THCP-d4	1793071-39-2	$C_{10}H_5D_4ClN_4S$	positive	257	190	/	35	21	/
Imidacloprid-d4	IMI-d4	1015855-75-0	$C_9H_6D_4ClN_5O_2$	positive	260	179	/	35	20	/
N-desmethyl-acetamiprid-$^{13}C_2{}^{15}N$	N-dm-ACE-$^{13}C_2{}^{15}N$	2484179-07-7	$C_7{}^{13}C_2H_9ClN_3{}^{15}N$	positive	212	126	/	50	25	/

* Product①was used for quantification, and Product②for confirmation; CV: cone voltage; CE: collision energy; MDL: method detection limit.

Table S2 Recoveries and precision of NEOs in urine samples of children (n=8).

No.	Compounds	Quantitative Internal Standards	Spiked at 0.1 ng/mL		Spiked at 1.0 ng/mL		Spiked at 4.0 ng/mL		Calibration Curve	R	Matrix Effect (%)
			Recovery%	RSD%	Recovery%	RSD%	Recovery%	RSD%			
1	IPP	ACE-d_3	75.5	8.4	89.4	4.9	90.5	4.1	y=2.796x+0.001	0.9986	88.6
2	IMIZ	IMIZ-d_4	74.5	7.8	87.4	4.2	96.0	1.8	y=0.262x+0.001	0.9994	83.3
3	CYC	IMI-d_4	82.5	10.2	74.2	8.0	85.6	7.5	y=0.469x+0.001	0.9979	98.0
4	NIT	NIT-d_3	104.5	4.5	68.7	3.5	78.9	5.5	y=0.197x+0.0001	0.9996	84.9
5	FLU	SUL-d_3	85.5	3.9	85.1	3.0	106.5	2.8	y=0.190x+0.0001	0.9999	100
6	SUL	SUL-d_3	86.5	7.3	79.1	7.2	90.1	8.6	y=0.052x+0.0003	0.9981	82.6
7	DIN	DIN-d_3	102.5	9.6	93.8	3.5	108.2	5.5	y=0.064x+0.0001	0.9984	106
8	THM	THM-d_3	89.5	5.2	88.5	3.7	94.5	4.2	y=0.259x-0.003	0.9994	106
9	ACE	ACE-d_3	96.5	3.6	91.4	3.3	100.9	4.6	y=0.239x+0.0006	0.9997	87.9
10	CLO	CLO-d_3	102.5	6.2	89.9	3.2	96.7	4.0	y=0.379x+0.0003	0.9996	85.5
11	THCP	THCP-d_4	89.5	4.4	86.5	5.0	91.4	3.0	y=2.025x+0.0007	0.9993	88.3
12	IMI	IMI-d_4	89.5	6.3	87.7	7.1	93.6	2.4	y=0.251x+0.0003	0.9998	93.2
13	FLO	THM-d_3	93.5	4.8	96.2	2.4	108.5	3.2	y=0.577x-0.005	0.9997	101
14	*N*-dm-ACE	N-dm-ACE-$^{13}C_2{}^{15}N$	92.5	7.0	91.5	4.8	103.3	3.8	y=0.236x+0.0001	0.9995	89.2
15	5-OH-IMI	IMI-d_4	80.5	6.4	73.6	3.8	72.8	1.8	y=0.128x-0.001	0.9995	91.9
16	THCP-AM	CLO-d_3	100.5	3.4	98.4	4.8	103.0	4.2	y=1.119x+0.0005	0.9997	90.1

Table S3 Reference dose (R*f*D) and relative potency factors (RPF) for NEOs.

Analyte	R*f*D[a] (mg/kg bw/day)	RPF	Ref.
ACE	0.071	0.80[b]	
CLO	0.0098	5.82[b]	
DIN	0.02	2.85[b]	
IMI	0.057	1.00[b]	
THM	0.006	9.50[b]	
THCP	0.004	14.25[b]	
NIT	/	3.62	[49]
FLO	/	1.00[d]	[50]
N-dm-ACE	0.071	0.80[c]	
5-OH-IMI	0.057	1.00[c]	
THCP-AM	0.004	14.25[c]	[14-16]
SUL	/	1.00[d]	
IMI_{eq}	0.057	1.00	[14]

a Dietary reference dose (R*f*D) obtained from USEPA (http://www3.epa.gov/pesticides/chem_search/cleared_reviews).

b Relative potency factor (RPF) was calculated based on the R*f*D of imidacloprid.

c The same RPFs with their parent compounds IMI, ACE, and THCP were used for 5-OH-IMI, N-dm-ACE, and THCP-AM because of lacking their own R*f*D.

d Relative potency factor (RPF) was set to the same R*f*D for IMI in current study, as their RfD values were not available.

Table S4 Excretion proportion values(*f*) of NEOs in urine.

Analyte	*f*	Pharmacokinetic dataoringin	Ref.
CLO	0.596[a]	Human	[58]
IMI	0.133[a]	Human	[58]
DIN	0.899[a]	Human	[58]
N-dm-ACE	0.586[a]	Human	[58]
ACE	0.48[a]	Human	[64]
SUL	0.51[a]	Human	[64]
5-OH-IMI	0.31[a]	Human	[64]
NIT	0.46[b]	Mice	[39]
THM	0.75[b]	Mice	[39]
THCP	0.0185[b]	Mice	[57]
FLO	0.51[C]		[38]
THCP-AM	0.0185[C]		

a based onthe human pharmacokinetic data;

b based on the mice pharmacokinetic data, due toa lace of human pharmacokinetic data;

c since no human or animal pharmacokinetic data were available, their urine excretion proportions were set to those of compounds with a similar chemical structure of parent compound. FLO were the same as SUL, THCP-AM was the same as THCP.

Table S5 Summary of mean (median) concentrations (μg/L) of NEOs in urine samples from this study with previous studies.

Participants	Sites	CLO	N-dm-ACE	THM	DIN	5-OH-IMI	FLO	SUL	IMI	NIT	ACE	THCP	THCP-AM	∑NEOs	Reference
Children	South China	5.89(1.93)	3.47(1.64)	1.85(0.79)	1.73(0.22)	0.77(0.11)	0.25(0.10)	0.56(0.06)	0.21(0.06)	0.11(0.03)	0.03(0.02)	0.01(0.003)	0.02(0.01)	14.9(8.63)	This study
Children	South China	1.7(0.71)	2.3(1.1)	1.2(0.3)	0.52(0.21)	0.89(0.36)	nd	nd	0.44(0.12)	nd	0.05(0.02)				[4]
Children	South China	1.42	0.72	0.57	nd	nd	nd	nd	0.79	0.29	< LOD	0.02	3.18		[45]
School children	East China	(< LOD)	(1.35)	(< LOD)	(< LOD)	(< LOD)	nd	nd	(< LOD)	(< LOD)	(< LOD)	(< LOD)		(3.00)	[40]
Children	Chongqing, China	5.18[a]	29.71[a]	3.88[a]	3.13[a]	nd	1.76[a]	1.48[a]	4.08[a]	3.06[a]	2.41[a]	3.05[a]	nd		[16]
Children	U.S.	(< LOD)	(0.21)	nd	nd	(< LOD)	nd	nd	(< LOD)	nd	(< LOD)	(< LOD)	nd		[15]
Middle school students	Chongqing, China	(5.12)[a]	(48.7)[a]	(3.11)[a]	(2.41)[a]	nd	(1.81)[a]	(1.53)[a]	(3.42)[a]	(3.06)[a]	(2.35)[a]	(2.24)[a]	nd	(88.1)[a]	[54]
University student	Zhejiang, China	0.26(0.12)	2.26(0.96)	0.17(0.05)	0.65(0.24)	0.38(0.08)	0.19(<LOD)	nd	0.09(<LOD)	0.10(<LOD)	0.05(<LOD)	0.02(<LOD)	0.02(<LOD)		[60]
Young adults	China	0.99(0.30)	0.92(0.35)	1.92(0.07)	0.79(0.26)	nd	nd	nd	0.04(0.02)	nd	0.05(0.01)	0.99(0.30)	nd	9.03(7.74)	[53]
University student	China	(0.19)	(0.48)	(0.09)	(0.16)	(2.08)	nd	nd	(0.09)	nd	(0.02)	(0.02)	nd	(5.60)	[56]
High school students	Guangzhou, China	(0.08)	(0.04)	(0.06)	(1.59)	(0.03)	nd	nd	(0.09)	nd	(0.03)	(0.004)	nd		[31]
Adults	Wuhan, China	0.83(0.29[a])	1.66(0.75)	0.55(0.21)	0.37(0.16)	nd	nd	nd	0.14(0.08)	nd	0.05(<LOD)	nd	nd	14.2(7.33)	[62]
General population	Nine countries	0.26(0.11)	nd	0.28(0.11)	3.05(0.68)	nd	0.07(<LOD)	0.01(<LOD)	0.19(0.13)	0.08(0.01)	0.01(<LOD)	nd	nd		[59]
General population	New York, USA	(0.24)	nd	(0.08)	nd	nd	(0.01)	(0.02)	(0.11)	(< LOD)	(< LOD)	(< LOD)	nd		[52]
General population	Six regions of China	(0.24)	nd	(0.08)	nd	(1.03)	nd	nd	(0.08)	nd	nd				[14]
General population	Rural Henan, China	0.18	0.42	0.26	nd	nd	nd	nd	0.75	0.26	0.17	0.30	0.31		[39]

General population	Shanghai, China	(0.001)	(0.006)	(0.002)	(0.001)	(0.001)	nd	nd	(0.020)	nd	(0.050)	(<LOD>	nd	(0.136) (IMIeq 0.249)	[46]
General population	Ten cities, China	nd	1.37(0.45)	nd	nd	2.63(1.02[a])	nd	nd	nd	nd	nd	nd	nd		[18]
Pregnant	Wenzhou, China	(0.45)	(1.33)	(<LOD)	(<LOD)	(<LOD)	nd	nd	(<LOD)	(<LOD)	(<LOD)	(<LOD)	nd	(3.96)	[25]
Pregnant	Japan	(0.14)	(0.34)	(0.05)	(0.22)	nd	nd	< LOD	< LOD	< LOD	< LOD	< LOD	< LOD		[47]
Pregnant	Guangdong, China	(0.10)	(0.83)	(0.07)	nd	(0.58)	nd	nd	(0.03)	nd	nd	nd	nd		[21]
Pregnant	Wuhan, China	(0.04)	(1.40)	(0.07)	nd	(0.64)	nd	nd	(0.04)	nd	(<LOD)	nd	nd		[55]
Pregnant	Tibetan, China	(< LOD)	(< LOD)	(< LOD)	(< LOD)	nd	nd	nd	(< LOD)	(< LOD)	(< LOD)	(< LOD)	nd	0.40	[39]
Pregnant	Jiangsu, China	(< LOD)	(0.25)	nd	(< LOD)	(0.33)	(< LOD)	(< LOD)	(< LOD)	(< LOD)	(< LOD)	nd	nd	(19.76)	[19]

[a] geometric mean creatinine concentrations (μg/g creatine); nd: not detected;

Table S6 Region-specific levels of NEOs in urine samples (μg/L).

	Rural					Urban					p
	Median	GM	Mean ± SD	Range	P95	Median	GM	Mean ± SD	Range	P95	
CLO	2.55	2.37	8.06 ± 30.06	<MDL–222	11.9	1.38	1.63	4.02 ± 8.29	0.170–49.9	30.1	<0.001***
N-dm-ACE	2.55	2.56	5.56 ± 10.8	<MDL–89.7	15.5	0.924	0.975	1.68 ± 2.13	0.052–12.8	7.12	<0.001***
THM	1.39	1.16	2.30 ± 2.78	<MDL–12.4	9.25	0.61	0.635	1.47 ± 2.38	<MDL–14.1	6.53	<0.001***
DIN	0.06	0.051	1.348 ± 4.95	<MDL–38.6	4.93	0.34	0.139	2.06 ± 4.51	<MDL–24.9	13.6	0.015*
5-OH-IMI	0.152	0.104	0.320 ± 0.489	<MDL–2.79	1.27	0.056	0.057	1.15 ± 5.89	<MDL–51.3	3.06	0.052
FLO	0.209	0.128	0.277 ± 0.270	<MDL–1.30	0.831	0.055	0.039	0.221 ± 0.456	<MDL–2.49	1.14	<0.001***
SUL	0.003	0.029	0.322 ± 0.585	<MDL–3.81	1.30	0.106	0.073	0.771 ± 2.60	<MDL–20.6	2.62	0.030*
IMI	0.058	0.044	0.115 ± 0.178	<MDL–1.17	0.345	0.057	0.055	0.297 ± 1.22	<MDL–11.3	0.738	0.672
NIT	0.003	0.008	0.053 ± 0.140	<MDL–0.825	0.357	0.077	0.056	0.167 ± 0.335	<MDL–3.00	0.510	<0.001***
ACE	0.015	0.017	0.039 ± 0.093	<MDL–0.661	0.142	0.016	0.012	0.023 ± 0.035	<MDL–0.30	0.068	0.818
THCP-AM	0.016	0.015	0.022 ± 0.017	<MDL–0.075	0.058	0.01	0.009	0.012 ± 0.009	<MDL–0.050	0.029	<0.001***
THCP	0.003	0.007	0.018 ± 0.037	<MDL–0.231	0.074	0.003	0.005	0.011 ± 0.025	<MDL–0.193	0.067	0.003**
∑m-NEOs	7.23	2.90	5.90 ± 10.8	0.009–90.4	15.8	1.24	1.30	2.84 ± 7.11	<MDL-64.1	8.22	<0.001***
$\sum_8$NEOs	9.91	9.30	17.8 ± 32.2	0.020–227	39.6	5.71	5.98	10.7 ± 16.1	<MDL–106	39.9	<0.001***
IMI_{eq}	37.8	38.5	78.8 ± 178	0.736–1315	184	24.2	26.4	48.0 ± 67.0	<MDL–390	208	0.006**
$\sum_{16}$NEOs	10.3	10.2	18.4 ± 32.2	0.614–227	39.7	7.02	6.95	11.0 ± 16.7	<MDL–107	45.1	0.002**

* $p < 0.05$ level (2-tailed); ** $p < 0.01$ level (2-tailed); *** $p < 0.001$ level (2-tailed).

Table S7 Spearman's correlation coefficient of NEOs

	5-OH IMI	NIT	SUL	DIN	THM	N-dm-ACE	ACE	CLO	IMI	FLO	THCP-AM	$\sum_8$NEOs	IMI_{eq}	$\sum_{12}$NEOs
5-OH IMI	1													
NIT	-0.087	1												
SUL	0.004	-0.143	1											
DIN	0.191**	0.267*	0.122	1										
THM	0.471**	-0.011	-0.044	0.051	1									
N-dm-ACE	0.368**	-	-0.153*	-0.004	0.340*	1								
ACE	0.057	0.036	-	0.140	0.137	0.362**	1							
CLO	0.432**	0.021	-0.082	0.120	0.716*	0.370	0.185*	1						
IMI	0.768**	-0.111	0.026	0.138	0.366*	0.164*	0.115	0.285*	1					
FLO	0.288**	0.076	-0.185*	0.079	0.387*	0.440**	0.307*	0.350*	0.182*	1				
THCP-AM	0.167*	-0.078	0.067	0.000	0.195*	0.176*	0.034	0.225*	-0.018	0.187	1			
$\sum_8$NEOs	0.490**	-0.023	-0.109	0.252*	0.589*	0.645**	0.286*	0.775*	0.306**	0.324	0.174*	1		
IMI_{eq}	0.451**	0.082	-0.052	0.256*	0.769*	0.420**	0.210*	0.893*	0.296**	0.346	0.204**	0.908**	1	
$\sum_{12}$NEOs	0.470**	0.008	-0.032	0.253*	0.588*	0.618**	0.262*	0.776*	0.292**	0.334	0.190**	0.984**	0.914*	1

** $p < 0.01$ level (2-tailed); * $p < 0.05$ level (2-tailed).

Table S8 Concentrations of NEOs with different demographic characteristics.

Analyte	Region	Age	BMI	Gender	Family income	Maternal educational attainment
CLO	-0.251***	-0.075	-0.105	0.089	-0.122	0.005
N-dm-ACE	-0.452***	-0.093	-0.133	0.020	-0.050	0.036
THM	-0.258***	-0.204**	-0.062	0.015	-0.108	-0.007
DIN	0.178*	-0.056	-0.088	-0.108	0.029	0.153*
5-OH IMI	-0.144	-0.151*	-0.095	0.051	-0.028	0.103
FLO	-0.305***	-0.100	-0.059	-0.016	-0.070	0.009
SUL	0.160*	-0.093	0.046	-0.052	-0.259***	0.078
IMI	0.029	-0.007	-0.066	0.045	-0.042	0.061
NIT	0.487***	0.114	0.015	0.030	0.151*	0.100
ACE	-0.017	0.084	0.015	0.002	0.101	0.060
THCP-AM	-0.266***	-0.089	0.187*	-0.007	-0.032	-0.003
∑m-NEOs	-0.393***	-0.115	-0.121	0.073	-0.073	0.026
$\sum_8$NEOs	-0.258***	-0.127	0.118	0.100	-0.151*	0.016
IMIeq	-0.202**	-0.129	-0.112	0.089	-0.159*	0.019
$\sum_{12}$NEOs	-0.232**	-0.122	-0.112	0.095	-0.170*	0.005
5-OH-IMI/IMI	-0.278***	-0.224**	-0.030	0.005	0.000	0.062
N-dm-ACE/ACE	-0.302***	-0.144	-0.115	0.034	-0.100	-0.058
THCP-AM/THCP	0.002	0.084	0.206**	0.154*	0.109	-0.082

* $p < 0.05$ level (2-tailed); ** $p < 0.01$ level (2-tailed); *** $p < 0.001$ level (2-tailed).

Table S9 Concentrations of NEOs in urine samples among school-aged children with different lifestyle habits.

**Analyte**	**Using cockroach killer**	**Using insecticide**	**Using mosquito coils**	**Engaging in farming**	**Second smoking**
CLO	-0.123	-0.102	0.061	-0.066	0.133
N-dm-ACE	-0.051	-0.058	0.143	0.057	0.122
THM	-0.102	-0.019	-0.011	-0.025	0.199**
DIN	0.043	-0.115	-0.028	-0.066	0.002
5-OH IMI	0.018	-0.111	-0.013	-0.004	0.114
FLO	-0.171*	-0.023	0.065	-0.009	0.161*
SUL	0.022	0.077	-0.118	0.079	-0.153*
IMI	0.036	-0.160*	0.032	-0.032	0.168*
NIT	0.095	-0.003	0.020	-0.070	-0.011
ACE	-0.098	-0.102	0.060	-0.049	0.127
THCP-AM	-0.056	-0.060	-0.052	-0.001	0.083
∑m-NEOs	-0.013	-0.073	0.129	0.047	0.117
$\sum_8$NEOs	-0.141	-0.145*	0.252	-0.027	0.118
IMIeq	-0.153*	-0.119	0.043	-0.060	0.141
$\sum_{16}$NEOs	-0.151*	-0.115	0.078	-0.005	0.113
5-OH-IMI/IMI	0.020	0.049	0.501	0.029	-0.033
N-dm-ACE/ACE	0.027	0.060	0.091	0.090	0.079
THCP-AM/THCP	0.001	-0.029	0.511	-0.043	0.023

* $p < 0.05$ level (2-tailed); ** $p < 0.01$ level (2-tailed); *** $p < 0.001$ level (2-tailed).

Table S10 Concentration ratios of m-NEOs/p-NEOs in urine samples.

	Median	Mean ± SD	GM	Range	P95
5-OH-IMI/IMI	1.85	4.14 ± 8.77	1.51	0.022–75.3	14.5
N-dm-ACE/ACE	1034	287 ± 601	106	2.64–6048	1041
THCP-AM/THCP	2.14	3.42 ± 3.60	2.01	0.078–24.9	10.4

Table S11 Effects of demographic characteristics on ratio of m-NEO/p-NEO.

	5-OH-IMI/IMI				N-dm-ACE/ACE				THCP-AM/THCP			
	**Median**	**Mean**	**P95**	***p***	**Median**	**Mean**	**P95**	***p***	**Median**	**Mean**	**P95**	***p***
**Regions**												
Urban	1.33	2.55	8.59	<0.001	66.5	198	924	<0.001	2.30	3.11	9.21	0.981
Rural	2.24	6.00	30.8		169	379	1855		1.82	3.79	11.5	
**Age (years)**												
9	2.27	6.01	35.3	0.002	144	327	1192	0.052	1.68	3.24	10.4	0.259
10	1.29	2.32	8.46		86.0	327	924		2.32	3.60	9.78	
**Gender**												
Boy	1.88	4.53	17.25	0.948	103	287	1009	0.644	1.77	3.06	9.78	0.038
Girl	1.81	3.61	8.59		104	274	1728		2.91	3.94	10.82	
**BMI**[*]												
Normal	1.88	3.90	16.40	0.686	111.2	301	1460	0.121	1.82	3.18	10.4	0.005
Abnormal	1.74	5.71	14.39		71.1	158	627		4.11	5.00	10.8	

Table S12 Summary of median EDI (μg/kg bw/day) of NEOs from this study with previous studies.

Participants	Site	CLO	N-dm-ACE	THM	DIN	5-OH-IMI	FLO	SUL	IMI	NIT	ACE	THCP	THCP-AM	∑NEOs	Reference
Children	Guangdong, China	0.127	0.117	0.046	0.009	0.013	0.008	0.005	0.017	0.002	0.001	0.008	0.026	0.696 (IMIeq 2.83)	This study
General population	Six regions of China	0.05	nd	0.13	nd	nd	nd	nd	0.10	nd	0.04	nd	nd	(IMIeq 3.31)	[14]
Children	China	2.90E-3	7.97E-4	2.30E-3	1.49E-4	1.44E-4	9.41E-5	nd	3.90E-3	1.37E-4	3.40E-3	3.06E-5	nd		[24]
Adults	China	1.40E-3	1.92E-4	5.65E-4	3.59E-5	3.48E-5	2.27E-5		9.50E-4	3.31E-5	8.30E-4	7.39E-6	nd		
University student	Guangdong, China	0.005	0.020	0.003	0.010	0.058	nd	nd	0.002	nd	0.001	0.001	nd	0.127 (IMIeq 0.235)	[56]
Young adults	Guangdong, China	0.038	0.040	0.02	0.05	nd	nd	nd	0.02	nd	0.001	0.005	nd	0.206 (IMIeq 0.526)	[53]
School children	East China	<LOD	nd	<LOD	<LOD	nd	nd	nd	<LOD	nd	0.07	<LOD	nd	0.13	[40]
Adults	Wuhan, China	/	/	/	nd	nd	nd	nd	/	/	/	nd	nd	0.08 (IMIeq 0.17)	[62]
Pregnant	Wenzhou, China	0.02	nd	<LOD	nd	nd	nd	nd	<LOD	<LOD	0.03	nd	nd	0.10	[25]
Middle school students	Chongqing, China	0.0175 (0.0170)[a]	nd	0.0085 (0.0087)[a]	0.005 (0.0040)[a]	nd	0.0074 (0.0041)[a]	0.0034 (0.0036)[a]	0.0547 (0.0576)[a]	0.0136 (0.0079)[a]	0.1767 (0.1668)[a]	0.2481 (0.2457)[a]	nd		[54]
Children	Japan	<LOD (0.0381)[b]	nd	<LOD (0.0076)[b]	<LOD (0.1380)[b]	nd	nd	nd	<LOD (0.1020)[b]	<LOD (0.0009)[b]	nd	<LOD (0.0033)[b]	nd	0.010 (0.350)[b]	[26]
Adults	Japan	<LOD (0.0239)[b]	nd	<LOD (0.0060)[b]	0.0559 (0.0039)[b]	nd	nd	nd	<LOD (0.0983)[b]	<LOD (0.0003)[b]	nd	<LOD (0.0008)[b]	nd	0.0092 (0.236)[b]	
Pregnant	Wuhan, China	0.0035	0.064	0.0036	nd	0.0323	nd	nd	0.002	nd	<LOD	nd	nd	0.157 (IMIeq 0.665)	[55]
Pregnant	Tibetan, China	<LOD	nd	<LOD	<LOD	nd	nd	nd	<LOD	<LOD	<LOD	<LOD	nd	0.030	[39]

[a] geometric mean value; nd: not detected; b mean value; /: not given;

Table S13 Non-carcinogenic risk (HQ) of NEO exposure in urine samples among school-aged children.

	Median	GM	Mean	Min	Max	P95
CLO	1.27E-02	1.29E-02	4.14E-02	5.80E-06	1.80E+00	8.25E-02
THM	7.63E-03	7.40E-03	1.67E-02	2.30E-05	1.69E-01	6.96E-02
AM-THCP	6.41E-03	6.26E-03	8.72E-03	1.28E-03	4.58E-02	2.41E-02
THCP	1.88E-03	3.11E-03	8.07E-03	8.79E-04	1.33E-01	3.97E-02
N-dm-ACE	1.65E-03	1.58E-03	3.70E-03	2.66E-06	1.08E-01	1.12E-02
DIN	4.63E-04	1.92E-04	4.02E-03	3.62E-06	7.86E-02	2.57E-02
IMI	3.00E-04	2.60E-04	1.13E-03	9.54E-06	6.46E-02	2.74E-03
5-OH-IMI	2.30E-04	1.59E-04	1.64E-03	3.77E-06	1.18E-01	3.51E-03
ACE	1.78E-05	1.67E-05	3.64E-05	6.93E-07	7.20E-04	9.89E-05
∑m-NEOs	1.01E-02	1.01E-02	1.41E-02	1.38E-03	1.34E-01	3.38E-02
$\sum_8$NEOs	3.83E-02	3.82E-02	7.67E-02	1.46E-03	1.82E+00	2.60E-01
IMI_{eq}	4.97E-02	5.08E-02	8.87E-02	3.64E-03	1.87E+00	2.75E-01
HI	4.69E-02	2.67E-02	8.54E-02	2.86E-03	1.83E+00	2.66E-01

Table S14 Concentrations, frequancy, toxicity, accumulation, persistence and ToxPi scores of NEOs.

Analyte	Concentration	Frequency	Toxicity	Accumulation	Persistence	ToxPi
CLO	1.00	1.00	0.37	0.49	0.04	0.581
THM	0.41	1.00	0.65	0.53	0.31	0.579
THCP-AM	0.01	0.87	1.00	0.60	0.10	0.516
N-dm-ACE	0.85	1.00	0.00	0.49	0.21	0.511
NIT	0.01	0.59	0.21	0.77	0.61	0.437
THCP	0.00	0.35	1.00	0.60	0.10	0.411
ACE	0.01	0.91	0.00	0.49	0.21	0.324
IMI	0.03	0.83	0.01	0.51	0.00	0.276
5-OH IMI	0.06	0.78	0.01	0.38	0.02	0.248
FLO	0.05	0.90	0.01	0.00	0.00	0.192
DIN	0.11	0.58	0.15	0.01	0.01	0.173
SUL	0.03	0.59	0.01	0.17	0.04	0.169
